# Clinical added value of 3D printed patient-specific guides in orthopedic surgery (excluding knee arthroplasty): a systematic review

**DOI:** 10.1007/s00402-025-05775-2

**Published:** 2025-03-03

**Authors:** Nick Kampkuiper, Romy ten Heggeler, Jorm Nellensteijn, Marjolein Brusse-Keizer, Gabriëlle Tuijthof, Maaike Koenrades, Femke Schröder

**Affiliations:** 1 Department of Biomechanical Engineering, University of Twente, Enschede, Netherlands; 2 Medical 3D Lab, Medisch Spectrum Twente, Enschede, Netherlands; 3 Department of Orthopedic Surgery, Medisch Spectrum Twente, Enschede, Netherlands; 4 Medical School Twente, Medisch Spectrum Twente, Enschede, Netherlands; 5 Health Technology & Services Research, Technical Medical Centre, University of Twente, Enschede, Netherlands; 6 Multi-Modality Medical Imaging (M3i) Group, Faculty of Science and Technology, Technical Medical Center, University of Twente, Enschede, Netherlands

**Keywords:** Patient-specific, 3D, Three-dimensional, 3D printing, Orthopedic surgery, Surgical planning, Systematic review, RCT, Clinical added value, Clinical outcome

## Abstract

**Introduction:**

Patient-specific guides (PSGs) provide customized solutions and enhanced precision. However, the question remains: does clinical evidence support the added value of PSGs? This study critically appraises, summarizes, and compares the literature to assess the clinical value of PSGs in orthopedic surgery.

**Materials and methods:**

PubMed and Embase were used to search for studies reporting on randomized controlled trials (RCTs) that compared the use of PSGs with a control group for an orthopedic intervention, excluding knee arthroplasty. The risk of bias was assessed using the Cochrane risk-of-bias tool (RoB 2). The clinical value was expressed as patient reported outcome measures (PROMs), complications, accuracy, surgery duration, blood loss, and radiation exposure. Relative and absolute differences were determined, and whether these were negative or positive for using PSGs.

**Results:**

From 6310 studies, 27 RCTs were included, covering various interventions. The studies' heterogeneity prevented meta-analysis. Six (22.2%) of the included articles scored low risk of bias. Significant differences in the benefit of PSGs were reported across all included metrics: 32.2% in PROMs, 22.7% in complications, 69.8% in accuracy, 42.1% in surgery duration, 46.7% in blood loss, and 93.3% in radiation exposure. No significant negative differences were found in any of the studies.

**Conclusion:**

PSGs generally show superior outcomes for accuracy and radiation exposure across multiple intervention types, while the reduction in complications was primarily significant in spinal fusion surgery. For PROMs, complications in other treatments, surgery duration, and blood loss, there may be clinical added value but future well-designed RCTs are needed to provide stronger evidence.

**Supplementary Information:**

The online version contains supplementary material available at 10.1007/s00402-025-05775-2.

## Introduction

In orthopedic surgery, patient specific guides (PSGs) could offer surgical guidance on pre-planned customized solutions, such as drilling, sawing, and (re-)positioning of bony structures [1]. The construction of a PSG begins with creating 3D bone models based on preoperative imaging, most commonly CT scans, to virtually plan the procedure. A digital 3D model is then designed to fit precisely onto a bony surface, incorporating the necessary functionality. These models are 3D printed and sterilized before surgery. During the operation, the bony surface is exposed, and the PSG is fitted onto the surface, enabling precision surgery.

3D printing is increasingly available for orthopedic surgeons. It is frequently used to create 3D printed anatomical models, offering surgeons supplementary insights into the nature of fractures or anatomy at relatively low cost. Studies have demonstrated that the use of 3D fracture models outperform conventional treatments across various outcomes [2–4]. PSGs can also potentially enhance the accuracy of specific treatments and provide customized solutions, but there is limited scientific evidence to support this. In maxillofacial and dental surgery, PSGs are more frequently utilized than in other specialties, and their use has been shown to result in superior outcomes [5, 6]. Just as 3D printed fracture models and PSGs in maxillofacial and dental surgery, PSGs could offer added value for various treatments in orthopedic surgery.

Despite the growing popularity in orthopedics, there is limited high level of evidence regarding the clinical added value. As described in the narrative review of Gauci et al., PSGs may offer several advantages including improved patient outcomes, reduced complication rates, increased accuracy, shorter surgery duration, decreased blood loss, and lower radiation exposure, compared to conventional interventions [1]. However, due to the narrative character, the clinical value of PSGs was not quantitatively assessed. One scoping review on 3D printing in orthopedic surgery, showed added value for 3D technology [7]. However, there remains a knowledge gap regarding PSGs specifically, as this review did not distinguish among types of 3D technology, e.g., PSGs, anatomical models, and virtual surgical planning. Furthermore, for total and unicompartimental knee arthroplasty, reviews have demonstrated minimal added value for PSGs [8–11]. This result could be explained by the fact that the standard knee arthroplasty operation kit is already somewhat patient-specific due to conventional sizing tools. However, for other types of orthopedic interventions where such kits are often lacking, PSGs may offer greater clinical value compared to conventional treatment.

This study aims to critically appraise, summarize, and compare the literature on randomized controlled trials (RCTs) that evaluate the clinical value of PSGs in orthopedic surgery compared to controls, excluding knee arthroplasty surgery.

## Materials and methods

This systematic review was performed in accordance with the Preferred Reporting Items for Systematic review and Meta‐Analyses (PRISMA) [12].

### Eligibility criteria

Studies were included that reported on RCTs in humans comparing the use of PSGs in orthopedic surgery with a control group. Studies investigating total or unicompartmental knee arthroplasty as well as phantom, animal, and cadaveric studies were excluded. This review focuses on recent literature, mainly because 3D technology has emerged and made substantial growth over the last decade. To ensure comprehensive coverage, studies of English origin and published in the past 15 years were considered.

### Search strategy

A systematic search was performed in PubMed and Embase in September 2023. A combination of three main search strings was used, including terms related to orthopedic surgery, surgical guides and methods of how these guides can be made or for what purposes they can be used (e.g., 3D printed or drilling, respectively). A filter was applied to include publications since 2008. Additionally, a specified RCT filter was applied [13]. Articles that mentioned total- or unicompartmental knee arthroplasty in the title were excluded. The complete search strategy can be found in Supplementary data I.

### Study selection

Two authors (N.K. and R.t.H.) independently screened the titles and abstracts according to the prespecified inclusion and exclusion criteria. After screening, the full text was read independently by two authors (N.K. and R.t.H.) to determine final inclusion. Discrepancies were discussed between reviewers and, if necessary, a third reviewer (F.S.) was involved to achieve consensus. Additionally, the references of included articles and some considered to be relevant reviews found during the screening process were hand‐searched to identify relevant studies that may have been overlooked by the search strategy.

### Risk of bias assessment and data extraction

All studies were independently assessed for risk of bias by two reviewers (N.K. and R.t.H.) using the revised Cochrane Risk of Bias 2 (RoB2) tool [14]. This tool scores on five domains: randomization process, deviation from intended interventions, missing outcome data, measurement of the outcome, and selection of the reported results, with scores of low risk, some concerns, and high risk of bias. The overall bias is determined by the worst outcome in a domain. Any discrepancies were discussed by the two reviewers, optionally with the third reviewer (F.S.). Both reviewers (N.K. and R.t.H.) independently extracted half of the data and cross-reviewed each other's extractions. In case of missing, unclear data, or data provided in figures, the author or journal was contacted by email (Table 1).

**Table 1:** Study Characteristics

Intervention	References	Country/Region	N	N Intervention group	N Control group	Procedure	Function PSG
Spinal fusion	Cecchinato [50]	Italy	29	14	15	Surgery for spinal deformity	Screw positioning
	Chen [51]	China	43	20	23	Lumbar pedicle screw fixation	Screw positioning
	Zhang [52]	China	40	20	20	Pedicle screw fixation for thoracolumbar fractures	Screw positioning
	Merc [53]	Slovenia	24	11	13	Pedicle screw positioning lumbar and sacral spine	Screw positioning
	Feng [54]	China	12	6	6	Cervical lateral mass screw fixation	Screw positioning
	Cui [55]	China	84	42	42	Sacral 2 alar iliac (S2AI) screws fixation	Screw positioning
	Merc [56]	Slovenia	19	9	10	Pedicle screw positioning lumbar and sacral spine	Screw positioning
Total hip arthroplasty (THA)	Wang [57]	China	104	Divided into four subgroups based Crowe’s classification for hip dysplasia (Crow I, II, III, IV)			
			I	21	21	Total hip arthroplasty in patients with four stages of hip dysplasia	Positioning acetabular component
			II	13	14		
			III	8	7		
			IV	10	10		
	Jin [58]	China	80	40	40	Total hip arthroplasty	Femoral stem positioning
	Zhang [59]	China	20	10	10	Metal-on-metal hip resurfacing arthroplasty	Positioning acetabular and femoral components
	Small [60]	United States	36	18	18	Total hip arthroplasty	Positioning acetabular component
	Zhang [61]	China	22	11	11	Total hip arthroplasty in patients with hip dysplasia	Reaming acetabulum
	Zhang [62]	China	53	23	30	Hip arthroplasty (total and hemi)	Positioning femoral stem
Anterior cruciate ligament (ACL) reconstruction	Zhu [63]	China	78	40	38	Anterior cruciate ligament reconstruction	Drilling tibial tunnel
	Liu [64]	China	41	22	19	Anterior cruciate ligament reconstruction	Drilling femoral and tibial tunnel
	Lan [65]	China	80	40	40	Anterior cruciate ligament reconstruction	Drilling femoral tunnel
High tibial osteotomy (HTO)	Gao [66]	China	39	16	23	High tibial osteotomy	Sawing and repositioning
	Zhu [67]	China	96	48	48	High tibial osteotomy	Sawing and repositioning
Total shoulder arthroplasty (TSA)	Boekel [68]†	Australia	47	24	23	Reversed total shoulder arthroplasty	Positioning glenoid component
	Hendel [69]	United States	31	15	16	Total shoulder arthroplasty	Positioning glenoid component
Percutaneous vertebroplasty	Hu [70]	China	36	18	18	Percutaneous vertebroplasty	Puncture assistance
	Chen [71]	China	97	47	50	Percutaneous vertebroplasty	Puncture assistance
Distal radius osteotomy	Buijze [72]	United States and Europe	37	20	17	Corrective Osteotomy Distal Radial Malunion	Sawing and repositioning
Distal humerus osteotomy	Hu [73]	China	35	16	19	Correction of cubitus varus deformity in children	Sawing assistance
Correction of lower limb deformities	Fan [74]	China	55	21	24	Correction of lower limb deformities in children	Drill assistance
Acetabular osteotomy	Ma [75]	China	22	11	11	Periacetabular osteotomy in acetabular dysplasia	Sawing assistance
Femoral neck fracture repair	Wang [76]	China	60	30	30	Femoral neck fracture repair	Screw positioning

The studies are ordered per intervention type and within an intervention on risk of bias assessment. † In the control group virtual surgical planning was used

### Outcome measures and analysis

The outcome measures of this systematic review included PROMs, complications, accuracy, surgery duration, blood loss, and radiation exposure. Accuracy metrics are included that compare the postoperative result quantitatively with the preoperative plan per individual, e.g., the positional (mm) or angular (°) deviation. Additionally, metrics were also included that describe the results of PSG use, such as the number of implants placed in safe zone or the number of insertions required to achieve the desired position. All six outcome measures are documented separately for the PSGs group and control group along with corresponding *p*-values (Table 2 and Supplementary data II). Due to the heterogeneity of orthopedic interventions and the variety of outcome measures (incl variety in follow-up), performing meta-analyses was deemed not appropriate. However, to summarize the outcomes and create a clear overview of the clinical value, the absolute changes (Δ) and relative changes (Δ%) of outcome measures have been displayed (Table 2 and Supplementary data II). These changes were determined by calculating the difference in mean or median values for continuous variables and by calculating the difference in percentages for discrete variables. If percentages were not explicitly stated, they were calculated based on the data provided by the study. For efficient interpretation of the results, the differences were described as positive or negative for PSGs in comparison to the controls. The notation used is: “–” for significant negative differences, “ + ” for significant positive differences, “(–)” for insignificant negative differences, “( +)” for insignificant positive differences, and “ = ” for no difference between the two groups. If it was unclear whether the difference was negative or positive for the use of PSG, it was marked as “?”. To assess the reliability of the studies, the overall risk of bias was calculated. Furthermore, to indicate if positive significant results originated more often from qualitative better studies with a lower risk of bias, the overall percentage of studies with low risk of bias was compared to the low risk of bias rate within all significant outcomes per outcome measure. In all tables and Fig. 2, the studies are ordered by intervention type, starting with those that have the most studies. Within each intervention type, studies are further ordered by their risk of bias score, as shown in Fig. 2.

**Table 2:** Study outcomes

Patient reported outcome measures (PROMs)									
Application	References	N	Patient outcome description	Value in intervention group	Value in control group	P-value	Absolute change (Δ)	Relative change (Δ%)	Positive or negative significant outcome for PSG
Total hip arthroplasty (THA)	Wang [57]	104	Divided into four subgroups based Crowe’s classification for hip dysplasia (Crow I, II, III, IV)						
		I	HHS pre-op	56.5 ± 10.4	55.8 ± 13.3	0.847	~	~	~
			HHS 3 m post-op	85.2 ± 3.6	84.5 ± 3.3	0.499	0.7	0.9	( +)
			HHS 1y post-op	88.4 ± 4.7	90.5 ± 3.8	0.088	− 2.0	− 2.2	(-)
		II	HHS pre-op	52.9 ± 13.1	54.3 ± 9.0	0.757	~	~	~
			HHS 3 m post-op	85.9 ± 4.4	84.3 ± 6.3	0.458	1.6	1.9	( +)
			HHS 1y post-op	89.8 ± 5.3	87.2 ± 3.3	0.170	2.5	2.9	( +)
		III	HHS pre-op	44.9 ± 17.1	43.0 ± 16.9	0.835	~	~	~
			HHS 3 m post-op	83.1 ± 5.1	77.7 ± 4.3	0.043*	5.4	7.0	+
			HHS 1y post-op	88.9 ± 5.5	82.4 ± 2.9	0.015*	6.4	7.8	+
		IV	HHS pre-op	40.3 ± 13.8	41.0 ± 11.2	0.902	~	~	~
			HHS 3 m post-op	71.9 ± 6.7	70.1 ± 7.1	0.566	1.8	2.6	( +)
			HHS 1y post-op	87.3 ± 3.5	83.2 ± 4.3	0.031*	4.1	4.9	+
	Jin [58]	80	VAS pre-op	6.4 ± 0.9	6.3 ± 1.1	0.657	~	~	~
			VAS 7d post-op	2.2 ± 0.6	2.3 ± 0.8	0.42	− 0.1	− 5.6	( +)
			VAS 4w post-op	1.6 ± 0.5	1.7 ± 0.8	0.41	− 0.1	− 7.5	( +)
			VAS 3 m post-op	1.4 ± 0.7	1.3 ± 0.6	0.857	0.1	+ 1.5	(–)
			VAS 6 m post-op	0.8 ± 0.6	0.9 ± 0.6	0.713	− 0.1	− 10.8	( +)
			VAS 12 m post-op	0.7 ± 0.5	0.7 ± 0.6	0.845	0.0	0.0	=
			HHS pre-op	48.9 ± 4.8	49.7 ± 5.6	0.536	~	~	~
			HHS 7d post-op	82.8 ± 5.0	76.8 ± 6.4	< 0.001*	6.0	7.7	+
			HHS 4w post-op	89.2 ± 2.8	85.9 ± 3.1	< 0.001*	3.3	3.8	+
			HHS 3 m post-op	93.9 ± 1.7	92.7 ± 1.8	0.003*	1.2	1.3	+
			HHS 6 m post-op	95.0 ± 1.8	94.5 ± 2.2	0.156	0.5	0.5	( +)
High tibial osteotomy (HTO)	Gao [66]	39	Clinical AKSS pre-op	57.5 ± 2.0	57.1 ± 2.4	0.616	~	~	~
			Clinical AKSS 3 m post-op	75.6 ± 7.3	70.2 ± 8.4	0.042*	5.5	7.8	+
			Clinical AKSS 6 m post-op	85.0 ± 6.1	80.2 ± 7.4	0.040*	4.8	6.0	+
			Clinical AKSS last follow-up	90.3 ± 6.2	89.4 ± 6.3	0.654	0.9	1.0	( +)
			Functional AKSS pre-op	58.8 ± 6.2	59.1 ± 7.6	0.870	~	~	~
			Functional AKSS 3 m post-op	70.9 ± 8.0	64.8 ± 9.0	0.034*	6.2	9.5	+
			Functional AKSS 6 m post-op	81.6 ± 4.7	80.4 ± 8.3	0.625	1.1	1.4	( +)
			Functional AKSS last follow-up	90.0 ± 6.3	89.6 ± 6.4	0.835	0.4	0.5	( +)
Total shoulder arthroplasty (TSA)	Boekel [68]†	47	CMS pre-op	30.2 ± 17.6	26.6 ± 16.3	0.48	~	~	~
			CMS 6 m post-op	68.6 ± 13.8	60.3 ± 16.3	0.06	8.3	13.8	( +)
			ASES score pre-op	33.7 ± 15.1	35.4 ± 10.5	0.64	~	~	~
			ASES score 6 m post-op	78.1 ± 16.0	75.3 ± 18.8	0.58	2.8	3.7	( +)
			OSS pre-op	24.3 ± 7.5	23.1 ± 6.1	0.57	~	~	~
			OSS 6 m post-op	40.7 ± 5.7	38.4 ± 6.6	0.20	2.3	6.1	(-)

Complications									
Application	References	N	Complication type	Number in intervention group	Number in control group	P-value	Absolute change (Δ)	Relative change (Δ%)	Positive or negative significant outcome for PSG
Spinal fusion	Cecchinato [50]	29	Malpositioned implants, Grade B–C (n, [%])	29 (9.8)	41 (16.9)	0.014*	− 7.1	− 42.0	+
			Malpositioned implants, Grade C (n, [%])	7 (2.4)	22 (9.1)	< 0.001*	− 6.7	− 73.6	+
			Dural lesion (n, [%])	1 (7.1)	1 (6.7)	NR	0.5	7.1	(−)
Total hip arthroplasty (THA)	Wang [57]	104	Divided into four subgroups based Crowe’s classification for hip dysplasia (Crowe I, II, III, IV)						
		III	DVT	1 (12.5)	0 (0)	NR	12.5	NaN	(−)
		IV	DVT	0 (0)	1 (10)	NR	− 10	− 100	(+)
			Transient paralysis of peroneal nerve (n, [%])	0 (0)	1 (10)	NR	− 10	− 100	(+)
			Transient paralysis of femoral nerve (n, [%])	0 (0)	1 (10)	NR	− 10	− 100	(+)
	Jin [58]	80	No complications occurred in both groups						
High tibial osteotomy (HTO)	Gao [66]	39	Lateral hinge fracture (n, [%])	0 (0)	2 (8.7)	NR	− 8.7	− 100	(+)
			Incisional exudation (n, [%])	1 (6.3)	0 (0)	NR	6.3	NaN	(−)
Total shoulder arthroplasty (TSA)	Boekel [68]†	47	Anterior dislocation (n, [%])	1 (4.1)	0 (0)	NR	4.1	NaN	(−)
			Subacromial bursitis (n, [%])	0 (0)	1 (4.3)	NR	− 4.3	− 100	(+)
			Sirveaux grade I + II scapular notching (n, [%])	2 (8.3)	3 (13.0)	0.73	− 4.7	− 36.1	(+)
	Hendel [69]	31	Nonoptimal implant type used during surgery (n [%])	1 (7)	10 (63)	< 0.001*	− 56	− 88.9	+
			Version or inclination malposition occurrences (n [%])	4 (13)	14 (44)	< 0.001*	− 31	− 221.4	+
			Malpositioned implants with > 10° deviation in version and/or inclination from the optimal preoperative plan (n [%])	4 (27)	12 (75)	< 0.01*	− 48.0	− 400	+
			Transient partial axillary nerve injury (n [%])	0 (0)	1 (5.9)	–	− 5.9	NaN	(+)

Accuracy									
Application	References	N	Method of measuring accuracy	Outcomes intervention group	Outcomes control group	P-value	Absolute change (Δ)	Relative change (Δ%)	Positive or negative significant outcome for PSG
Spinal fusion	Cecchinato [50]	29	Implants placed in safe zone, grade 0-A (n [%])‡	268 (90.2)	202 (83.1)	NR	7.1	8.5	(+)
Total hip arthroplasty (THA)	Jin [58]	80	Absolute leg length discrepancy (mm)	1.3 ± 1.0	4.7 ± 2.6	< 0.001*	− 3.5	− 73.5	+
			Leg length discrepancy ≤ 5 mm (n, [%])	40 (100)	28 (70)	< 0.001*	30	42.9	+
			Absolute femoral stem offset (mm)	3.0 ± 1.6	6.0 ± 2.7	< 0.001*	− 3.0	− 49.8	+
			Absolute femoral stem offset ≤ 5 mm (n, [%])	36 (90)	27 (67.5)	0.014*	22.5	33.3	+
			Absolute femoral stem anteversion (°)	3.5 ± 1.1	6.9 ± 2.3	< 0.001*	− 3.3	− 48.5	+
			Absolute femoral stem varus/valgus (°)	0.8 ± 0.4	2.4 ± 1.3	< 0.001*	− 1.5	− 64.4	+
High tibial osteotomy (HTO)	Gao [66]	39	Absolute difference from designed target						
			Weight-bearing line ratio (AU)	2.0 ± 1.8	5.4 ± 4.4	0.002*	− 3.5	− 63.7	+
			Hip-knee-ankle angle (°)	1.1 ± 0.9	2.3 ± 2.0	0.018*	− 1.2	− 50.7	+
			Medial proximal tibial angle (°)	1.0 ± 0.6	1.5 ± 1.0	0.068	− 0.5	− 31.8	(+)
			Correction angle (°)	0.7 ± 0.6	0.6 ± 0.4	0.912	0.0	3.1	(−)
			Posterior tibial slope angle (°)	1.3 ± 1.3	2.0 ± 2.0	0.244	− 0.1	− 34.0	(+)
Total shoulder arthroplasty (TSA)	Boekel [68]†	47	Guidewires placed within 2 mm of the planned position in superior/inferior plane (n, [%])	22 (91.7)	14 (14.0)	0.01*	30.8	50.6	+
			Guidewires placed within 2 mm of the planned position the AP plane (n, [%])	20 (83.3)	18 (78.3)	0.66	5.1	6.5	(+)
	Hendel [69]	31	Deviation in total offset (mm)	2.4 ± 1.6	3.4 ± 1.8	0.11	− 1.0	− 29.4	(+)
			Anteroposterior offset (mm)	1.0 ± 0.9	1.9 ± 1.4	0.06	− 0.9	− 47.4	(+)
			Medial–lateral offset (mm)	1.0 ± 0.9	1.9 ± 1.0	0.012*	− 0.9	− 47.4	+
			Superior-inferior offset (mm)	2.0 ± 1.5	2.3 ± 2.1	0.64	− 0.3	− 13.0	(+)
			Deviation in version (°)	4.3 ± 4.5	6.9 ± 4.4	0.11	− 2.6	− 37.7	(+)
			Deviation in inclination (°)	2.9 ± 3.4	11.6 ± 7.0	< 0.0001*	− 8.7	− 75.0	+
			Deviation in roll (°)	6.5 ± 5.1	10.2 ± 9.7	0.13	− 3.7	− 36.3	(+)
			Deviation from plan least retroverted (°)	7.0 ± 5.4	3.2 ± 2.1	0.14	3.8	118	(−)
			Deviation from plan most retroverted (°)	1.2 ± 2.0	10.0 ± 4.4	< 0.001*	− 8.8	− 88.0	+

Surgery duration									
Application	References	N	Description duration	Value in intervention group	Value in control group	P-value	Absolute change (Δ)	Relative change (Δ%)	Positive or negative significant outcome for PSG
Spinal fusion	Cecchinato [50]	29	Total surgery duration (min) in mean	422	423	> 0.05	− 1	− 0.2	(+)
Total hip arthroplasty (THA)	Wang [57]	104	Divided into four subgroups based Crowe’s classification for hip dysplasia (Crow I, II, III, IV)						
		I	Total surgery duration (min)	27.1 ± 3.4	24.6 ± 3.4	0.053	2.5	10.3	(−)
		II	Total surgery duration (min)	33.0 ± 6.5	33.1 ± 4.4	0.994	− 0.1	− 0.1	(+)
		III	Total surgery duration (min)	42.3 ± 4.2	50.0 ± 1.5	0.001*	− 7.7	− 15.5	+
		IV	Total surgery duration (min)	61.4 ± 14.4	70.5 ± 12.1	0.151	− 18.8	− 12.8	(+)
	Jin [58]	80	Total surgery duration (min)	78.4 ± 16.4	74.2 ± 13.2	0.583	4.2	5.7	(−)
High tibial osteotomy (HTO)	Gao [66]	39	Total surgery duration (min)	109.4 ± 20.8	131.7 ± 29.9	0.014*	− 22.3	− 16.9	+
Total shoulder arthroplasty (TSA)	Boekel [68]†	47	Total surgery duration (min)	78.4 ± 16.3	74.8 ± 10.3	0.42	3.6	4.8	(−)

Blood loss									
Application	References	N	Description duration	Value in intervention group	Value in control group	P-value	Absolute change (Δ)	Relative change (Δ%)	Positive or negative significant outcome for PSG
Total hip arthroplasty (THA)	Wang [57]	104	Divided into four subgroups based Crowe’s classification for hip dysplasia (Crow I, II, III, IV)						
		I	Blood loss (mL)	333.3 ± 149.4	328.6 ± 133.8	0.914	4.7	1.4	(−)
		II	Blood loss (mL)	361.5 ± 122.7	382.1 ± 156.4	0.706	− 20.6	− 5.4	(+)
		III	Blood loss (mL)	412.5 ± 83.5	435.7 ± 102.9	0.643	− 23.2	− 5.3	(+)
		IV	Blood loss (mL)	660.0 ± 206.6	875.0 ± 173.6	0.022*	− 215	–2 4.6	+
	Jin [58]	80	Intraoperative blood loss (mL)	435.8 ± 73.3	427.5 ± 69.8	0.395	8.3	1.9	(−)
			Total blood loss (mL)	711.3 ± 159.5	680.6 ± 148.2	0.528	30.7	4.5	(−)
High tibial osteotomy (HTO)	Gao [66]	39	Intraoperative blood loss (mL)	50.6 ± 14.4	97.8 ± 67.4	0.003*	− 47.2	− 48.2	+

Radiation exposure									
Application	References	N	Description duration	Value in intervention group	Value in control group	P-value	Absolute change (Δ)	Relative change (Δ%)	Positive or negative significant outcome for PSG
Spinal fusion	Cecchinato [50]	29	C-arm fluoroscopy shots (n)	11 ± 9.87	47.5 ± 15.3	0.001*	− 36.5	− 77	+
			DAP (cGycm^2^)	133.5 ± 59.6	473.3 ± 448.3	0.001*	− 339.8	− 71.8	+
			Fluoroscopy images (n)	13.2 ± 8.7	32 ± 20.9	0.001*	− 18.8	− 58.7	+
			Exposure time (s)	9.4 ± 2.9	28.3 ± 27.7	NR	− 19.0	− 66.8	+
High tibial osteotomy (HTO)	Gao [66]	39	Radiation exposure (n)	18.5 ± 4.8	28.22 ± 4.28	0.003*	− 9.7	− 34.4	+

Values are given in means with standard deviation unless indicated otherwise. Absolute change (Δ) and relative change (Δ%) are based on mean or median values in the case of continuous variables. In the case of discrete variables, these are based on percentages. If authors used more than one decimal place in their study, values are rounded to one decimal place unless rounding results in zero. To calculate the absolute and relative change the unrounded values are used. “–” for significant negative differences, “ + ” for significant positive differences, “(–)” for insignificant negative differences, “( +)” for insignificant positive differences, and “ = ” for no difference between the two groups. “?” was used when it was unclear whether a result was positive or negative for the use of PSG. ‡This article performed an intention-to-treat and per protocol analysis but in this table we show the results of the intention-to-treat analysis. † In the control group virtual surgical planning was used. ~ Should not be determined because this is a preoperative measurement. * Indicates a statistical significant difference. Abbreviations: American Knee Society Score (AKSS), American Shoulder and Elbow Surgeons (ASES), Anterior Cruciate Ligament (ACL), Arbitraty Unit (AU), Constant Murley Score (CMS), Deep Venous Trombosis (DVT), Dose Area Product (DAP), Harris Hip Score (HHS), Not Applicable (NA), Not Reported (NR), Not a Number (NaN), Oxford Shoulder Score (OSS), Visual Analogue Score (VAS)

## Results

### Search and RCT characteristics

**Fig. 1:**
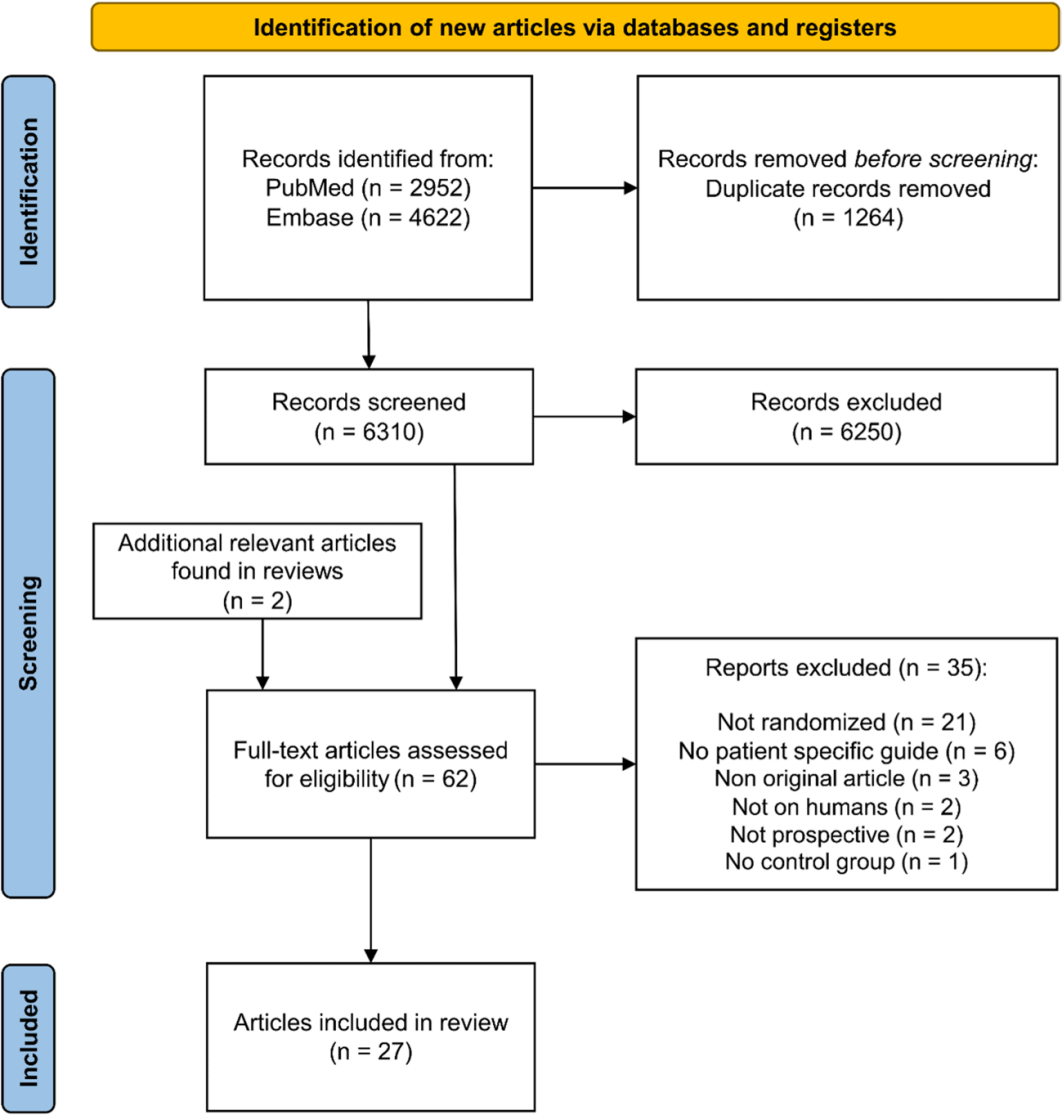
Flowchart of the screening and selection process

In the initial search, 7574 articles were identified. After removing duplicates, 6310 remained for title and abstract screening (Fig. 1). After screening, 6250 articles were excluded, and two potentially relevant articles were identified from reviews, resulting in 62 eligible studies for full-text screening. Thirty-five of these articles were excluded for the following reasons: twenty-one were not randomized [15–35], six did not use a PSG [36–41], three were non-original articles [42–44], two were not conducted on humans [45, 46], two were not prospective [47, 48], and one did not have a control group [49] (Fig. 1.). So, twenty-seven studies met the inclusion criteria. The references of the included studies revealed no relevant new publications. All included studies and characteristics are presented in Table 1 [50–76]. Seven studies reported on screw positioning for spine surgery [50–56]. Six studies reported on aiding implant positioning in total hip arthroplasty (THA) [57–62]. Three studies investigated guided drilling for anterior cruciate ligament (ACL) reconstruction [63–65]. Two studies focused on the sawing and repositioning in high tibia osteotomy (HTO) [66, 67]. Two studies focused on the positioning of the glenoid component in total shoulder arthroplasty (TSA) [68, 69]. Two studies investigated a puncture assisting device for percutaneous vertebroplasty [70, 71]. One study reported on sawing and repositioning of a distal radius malunion [72]. One study aided in sawing a distal humerus with a cubitus varus deformity [73]. One study assisted in drilling for the correction of lower limb deformities [74]. One study assisted in sawing during a periacetabular osteotomy [75]. Lastly, one study guided screw positioning for femoral neck fracture repair [76].

### Risk of bias assessment

**Fig. 2:**
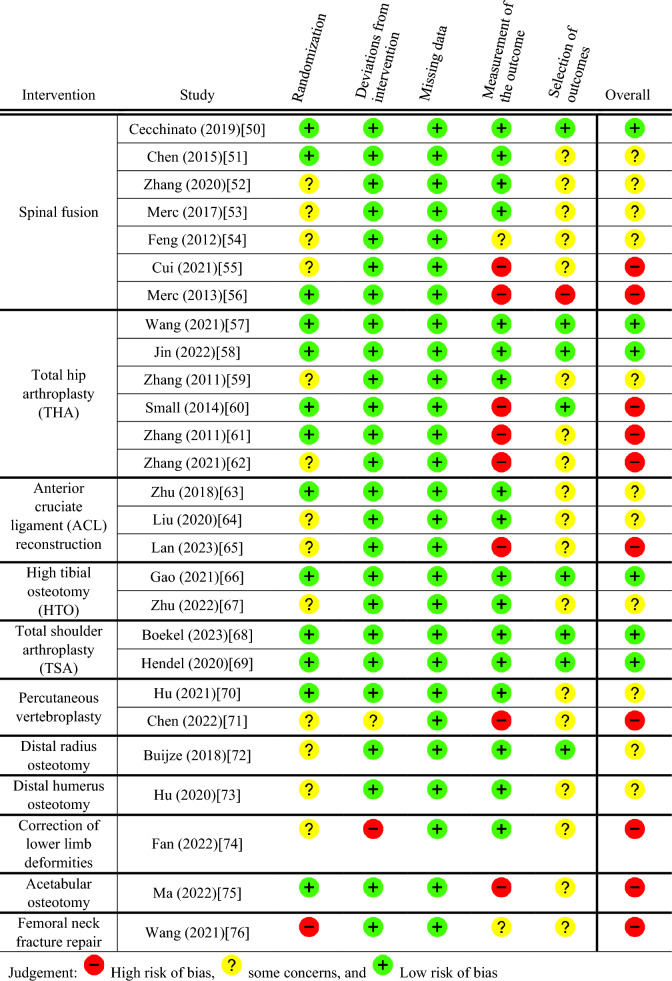
Risk of Bias assessment using Rob 2 tool. The studies are ordered per intervention type and within an intervention on risk of bias assessment

An overview of the risk of bias assessment is shown in Fig. 2. Six (22.2%) of the included articles scored a low risk of bias [50, 57, 58, 66, 68, 69]. Twenty-one (77.8%) of the included studies scored unclear or high risk of bias for at least one domain of the RoB2 tool. Eleven (40.7%) scored unclear [51–54, 59, 63, 64, 67, 70, 72, 73], and ten (37.0%) scored high risk of bias [55, 56, 60–62, 65, 71, 74–76].

### Outcome measures

The outcome measures of the studies with low risk of bias are shown in Table 2. A complete overview of the outcome measures of all studies can be found in Supplementary data II.

#### Patient reported outcome measures (PROMs)

PROMs were assessed in 18 (66.7%) studies, with some studies using multiple outcomes at multiple time points. Therefore, 87 measures were assessed: 27 (31.0%) were significantly positive for the PSG group [52, 57, 58, 66, 67, 71], 41 (47.1%) were insignificantly positive [52–55, 57, 58, 63–68, 72, 73, 75, 76], three (3.4%) showed no difference between groups [58, 71, 72], and 16 (18.4%) were insignificantly negative [57, 58, 65, 68, 70, 72] (Table 2).

#### Complications

Twenty-four (88.9%) studies reported the occurrence or absence of complications. Six of these reported no complications in either group [54, 58, 63, 65, 73, 75]. The remaining 18 studies reported complications 44 times, of which ten times (22.7%) significant reductions was reported [50, 52, 53, 55, 56, 69, 76], with absolute reductions in complications ranging from 6.7% to 56%. Remarkably, 6 out of these 10 significant reductions were observed in spinal fusion [50, 52, 53, 55]. Twenty-four complications (54.5%) were reported as insignificant reductions [51, 53, 55–57, 60, 66, 68–72, 74], three (6.8%) had the same number of complications in both groups [55, 67] and seven (15.9%) were insignificantly negative [50, 53, 57, 64, 66, 68, 71] (Table 2).

#### Accuracy

Out of the 27 articles included, twenty (74.1%) reported one or more objective accuracy outcomes, totaling 63 outcomes: 44 (69.8%) outcomes indicated significantly positive results for accuracy when using PSG compared to the control procedure[52, 54–56, 58–64, 66–69, 72, 73, 76], 11 (17.5%) were insignificantly positive[50, 56, 66, 68, 69], one (1.6%) showed no difference [65], four (6.3%) were insignificantly negative[60, 66, 69, 72], and three (4.8%) were unclear[65] (Table 2). In one article, some accuracy outcomes were shown in figures [68]. The authors were contacted but no response was obtained. Therefore, the data was denoted as not reported.

#### Surgery duration

The surgery duration was reported in 16 (59.3%) studies. One study had four different subgroups [57], resulting in 19 assessments of duration. In eight (42.1%) times, the duration was significantly decreased [52, 57, 59, 66, 70, 71, 74, 75], with relative time savings ranging from 15.4% to 30.2%. In five (26.3%) assessments the duration was insignificantly decreased [50, 54, 56, 57], and in six (31.6%) assessments the duration was insignificantly increased [57, 58, 60, 61, 68, 72] (Table 2).

#### Blood loss

Nine articles (33.3%) reported on blood loss with 15 different outcomes. Seven (46.7%) showed a significant decrease in blood loss related outcome measures [52, 57, 59, 66, 75] with relative decreases in blood loss ranging from 14.5% to 48.2%. Three measures (20.0%) showed an insignificant decrease [57, 61], and five (33.3%) showed an insignificant increase [54, 57, 58, 60] (Table 2). Eighteen (66.7%) articles did not report blood loss [50, 51, 53, 55, 56, 62–65, 67–74, 76].

#### Radiation exposure

Radiation exposure was reported in nine (33.3%) studies. In eight (29.6%) studies, it was not applicable [57–62, 68, 69], and in ten (37.0%) studies [53–56, 63–65, 67, 73, 76], it was not reported. In total, 15 outcomes were assessed: 14 (93.3%) measures showed significant decrease [50–52, 66, 70–72, 75] and one (6.7%) showed an insignificant decrease in radiation exposure [74] (Table 2).

#### Positive significance and risk of bias

The percentage of positive significant outcomes originating from studies with a low risk of bias was 33.3%, 50.0%, 23.8%, 25.0%, 28.6%, and 33.3% for PROMs, complications, accuracy, surgery duration, blood loss, and radiation exposure, respectively.

## Discussion

This systematic review aimed to critically appraise, summarize, and compare the literature on RCTs evaluating the clinical added value of PSGs in orthopedic surgery compared to conventional treatments. This review provides evidence that PSGs generally show superior outcomes for accuracy and radiation exposure across various intervention types, and specifically reductions of complications were primarily observed in spinal fusion (Table 2 and Supplementary data II). Other outcome measures, including PROMS, complications in other interventions than spinal fusion, surgery duration and blood loss, may also be superior, though the evidence for these measures is less strong. Furthermore, no significant negative effects for the use of PSGs were reported in any of the RCTs.

Thirty-one percent of the PROMS outcomes (27 out of 87) showed that PSGs were significantly better than conventional treatment [52, 57, 58, 66, 67, 71]. Remarkably, the rate of positive but insignificant results was relatively high at 47.1% (41 out of 87). This may suggest that studies lacked statistical power or that PSGs offer limited added value for certain interventions. Note, that the time intervals of the measurements varied considerably between studies; some studies had solely two intervals [54, 63, 68] (e.g., preoperative and 6 months postoperative [68]), while others included multiple intervals [52, 58, 66] (e.g. preoperative, 1 day, 7 days, 1 month, 3 months [52]). Some studies found a significant difference shortly after the surgery but no significant difference at later follow up measurements [52, 52, 58, 58, 66, 67, 67, 71]. It is debatable whether intervals shortly after surgery (e.g. 1 day, 7 days, 1 month) are clinically relevant, as the ultimate goal is to achieve superior long-term clinical outcomes. Therefore, to optimize the assessments of PROMs in future studies, it is recommended to standardize the intervals for each intervention type to preoperative, 3 months postoperative, 6 months, and 1 year, similar to the included studies on distal radius osteotomies [68] and HTOs [67]. To properly assess long term clinical outcome, 2 and 5 year follow up should be included in follow up studies. Furthermore, there is difference between specific types of PROMs that were used. This is of course depending on the type of surgery but even within the same intervention type different scores were used, such as American Knee Society Score (AKSS), the International Knee Documentation Committee (IKDC) score, and Lysholm Knee Score. This variability makes it difficult to compare studies and highlights the need for standardized PROMs per intervention type. Good practice was seen in studies on hip-related surgeries, as the Harris Hip Score (HHS) was consistently used in all studies reporting PROMs for such surgeries [57, 58, 75, 76].

There is evidence that PSGs can reduce the risk of complications. Specifically, there was a significant difference between the PSG and control groups in 22.7% (10 out of 44) of the complication measures [50, 52, 53, 55, 56, 69, 76], with absolute reductions in complications ranging from 6.7% to 56%. Six out of 10 significant differences were found in spinal fusion surgery [50, 52, 53, 55]. This indicates that for this type of surgery, where the complication rate is relatively high, PSGs can help reduce complications. Similar to the PROMs, the amount of positive but insignificant complications outcomes was substantially higher compared to the negative but insignificant outcomes. Again, we can argue that studies lack the statistical power to detect significant differences, as 63% of the included studies mention sample size as a study limitation [47, 49–51, 54, 55, 57, 60, 61, 63, 65, 66, 71, 73–76].

Accuracy was frequently assessed (20 studies) with results indicating that PSGs can significantly improve accuracy in 69.8% (44 out of 63) of the outcomes across all interventions [52, 54–56, 58–64, 66–69, 72, 73, 76]. Similarly to PROMs, accuracy metrics should be standardized for each intervention type. Ideally, these metrics should be based on absolute 3D positional and angular deviations between preoperative plan and outcomes, typically determined on postoperative CT imaging [58, 66, 69, 77, 78]. Five included studies determined the relative difference instead of absolute, which may overestimate the accuracy of a PSG [55, 56, 60–62]. Therefore, these studies scored high risk of bias in the fourth domain “Measurement of the outcome”. One article compared mean postoperative implant position metrics, such as the femoral anteversion angle, between the PSG group and control group [57]. Because this approach does not overestimate the accuracy of the PSG, this did not negatively affect the risk of bias assessment. However, this irrelevant metric was not included in the review.

The surgery duration was significantly decreased in 42.1% (8 out of 19) of the outcomes [52, 57, 59, 66, 70, 71, 74, 75], with the use of PSGs resulting in relative time savings ranging from 15.4% to 30.2% compared to the control group. Although it is not the most clinically relevant outcome measure, it might be of added value because shorter surgery duration can reduce the risk of infection [79] and may allow for more surgeries to be performed in a day. A decrease in surgery duration may be underestimated due to the learning curve associated with implementing PSGs. On the other hand, for certain applications, surgery duration might increase due to the need for sufficient bone exposure for proper guide placement.

Blood loss was the least reported outcome measure, yet 46.7% (seven out of 15) outcomes showed a significant decrease in blood loss [52, 57, 59, 66, 75]. This indicates that PSGs can reduce blood loss, possibly in close relation with the reduced surgery duration, as some studies who showed significant decrease in blood loss also reported significant decrease in surgery duration [52, 66, 75].

PSGs can clearly reduce the radiation exposure. In the results (Table 2) is shown that nearly all radiation exposure outcomes showed a significant radiation decrease, with only one [74] of the 15 [50–52, 66, 70–72, 75] outcomes showing an insignificant positive effect (Table 2). This reduction is likely due to the decreased need for additional fluoroscopic guidance, as the PSGs provide the necessary guidance, resulting in lower radiation exposure not only for the patient but also for the surgical team. Depending on the type of surgery, a preoperative scan may already be part of standard care. However, if an additional CT scan is required due to the use of a PSG, this could result in higher overall radiation exposure.

Radiation exposure shows the highest positive significance rate of 93.9%, though it was assessed less frequently compared to accuracy. Therefore, we assume that the evidence for added value of PSG is the strongest in terms of accuracy.

Two other reviews regarding 3D technology also found that 3D printed devices (not exclusively PSGs) are clinically effective [7, 80]. However, these reviews did not solely focus on PSGs [7] or orthopedics [80]. In oral and maxillofacial surgery studies have reported on PSGs clinical value in particular, which align with the findings of the present review. In a meta-analysis for jaw reconstructions, PSGs were associated with significant better aesthetic outcomes and reduced surgery duration of 21.2% (95% CI: 10–33%) [6]. These findings are consistent with some of the PROMs and surgery duration outcomes (significant findings ranging from 15.4% to 30.2%) in this review. Furthermore, an RCT that investigated the accuracy for PSGs in dental implantations, found a significantly better accuracy for the PSG group compared to the freehand control group, 3.04° ± 1.5 and 7.03° ± 3.44, respectively [5]. Other outcome measures, such as complications and blood loss are neither frequently investigated, nor show significant differences in oral and maxillofacial surgery [5, 6, 81]. These measures have been better assessed in orthopedic applications as has become apparent in this review. Though, it seems that, when larger studies are conducted with outcome measures standardized per intervention type, superior outcomes will be more consistently detected in orthopedic surgery similar to the studies that were performed in oral and maxillofacial surgery [5, 6, 81].

In Table 2, which contains the study characteristics, it is shown that solely 25.9% of the studies were conducted in the Western world. This is remarkably low considering the high standard of healthcare and research in these regions. This difference could be attributed to the Medical Device Regulation (MDR) legislation in the European Union [82] and the significant amount of time, money, and effort involved in conducting RCTs.

It should be noted that the overall low risk of bias rate was relatively low (22%). However, the percentage of positive significant outcomes originating from studies with low risk of bias were 33.3%, 50.0%, 23.8%, 25.0%, 28.6%, and 33.3% for PROMs, complications, accuracy, surgery duration, blood loss, and radiation exposure, respectively. This means that the low risk of bias studies, i.e., the higher quality studies, reported positive significant outcomes more frequently compared to studies with unclear and high risk of bias. In other words, the studies with unclear or high risk of bias do not seem to report significant findings more frequently.

The strength of this systematic review lies in its comprehensive overview of RCTs investigating the use of PSG in orthopedic surgery for multiple outcome measures. By focusing exclusively on RCTs, it ensures reliable comparisons with control groups. However, the restriction to RCTs can also be seen as a limitation, as it excludes case–control studies, such as for tumor resections [83] or HTOs [84]. In some cases, it might be more ethically appropriate to conduct a case–control study, especially when the new method is already considered superior. Nonetheless, we chose to include only RCTs because randomization, controlled and prospectively gathered data provides higher level of evidence. There may be publication bias, as studies that did not demonstrate added value for PSG are less likely to be published. Observer bias could also affect intraoperative outcomes, such as surgery duration or blood loss, since it is impossible to blind the surgeon. Another limitation is the heterogeneity of the included studies, limiting the feasibility of conducting a meta-analysis. This heterogeneity also affects the outcome measures, making it difficult to compare different guides for the same intervention. Furthermore, the limited number of trials within each subspecialty makes it difficult to draw conclusions within specific subspecialties, however this review gives a good general sense of patient specific guides in orthopedic surgery. Finally, it is important to note that significant differences in outcome measures do not necessarily result in clinically relevant differences. For example, Jin et al., observed a significant decrease in absolute femoral stem varus/valgus deviation from 2.4 ± 1.3° in the control group to 0.8 ± 0.4° (*p* < 0.001) in the PSG group, representing a mean reduction of 1.6° in varus/valgus deviation (Table 2) [58]. However, this difference may not be clinically relevant for this particular intervention.

The clinical implications based on this review suggest that the use of PSGs should be more widely adopted in orthopedic practice. Furthermore, we would plea for research to standardize outcome measures and follow up intervals to objectively compare conventional treatment with various PSG designs for specific procedures. In the included articles, the costs regarding the use of PSGs were barely mentioned. However, the costs that were mentioned for materials and total expenses (it is unclear what exactly these costs consist of) varied between 30 dollars and 840 euros [53, 58, 61, 63, 64], which may be dependent on local factors such as availability, requirements due to regulations, personnel costs, etc. The cost prevention associated with the use of PSGs, for example through a reduction in complications, was never mentioned. However, we suggest that future studies should also investigate the cost-effectiveness, as the broader implementation of PSGs is likely influenced by their financial viability. Additionally, while this review focused on comparing PSGs with conventional treatments, other technologies with similar goals, such as stereotactic navigation or augmented reality (AR), could also be included in comparisons to determine the most (cost) effective treatment method. For example, one of the included articles compared a PSG, a navigated technique, and the conventional treatment in HTO [67].

Although surgeons can accurately recreate a preoperative 3D plan using PSGs, navigation systems or AR, the optimal physiological, anatomical or mechanical 3D plan often remain unclear. For example the ideal patient-specific position of an implant can be unknown, while this could have effect on patient outcome. Therefore, future research should focus not only on the implementation and evaluation of PSGs, but also on determining the ideal implant positions or bone corrections to achieve best patient outcomes in orthopedic surgery.

## Conclusion

This systematic review summarizes the current literature on RCTs, evaluating the clinical added value of PSGs in orthopedic surgery, with exclusion of PSGs used in knee arthroplasty surgery. The findings demonstrate that PSGs often outperform conventional treatment in terms of accuracy and radiation exposure across multiple intervention types. Additionally, PSGs seem to reduce complications, particularly in spinal fusion surgery. Therefore, PSGs offer clinical value and should be more widely adopted in orthopedic practice. For the other outcome measures, including PROMs, complications in other interventions than spinal fusion surgery, surgery duration, and blood loss, there is moderate to limited evidence that PSGs are superior. To draw more definitive conclusions on the added value of PSGs, future research with well-designed RCTs are needed to provide stronger evidence.

## Supplementary Information

Below is the link to the electronic supplementary material.

Supplementary file 1 (DOCX 23 KB)

Supplementary file 2 (DOCX 298 KB)

## Data Availability

No datasets were generated or analysed during the current study.

## Abbreviations

AKSS: American knee society score
ASES: American shoulder and elbow surgeons
ACL: Anterior cruciate ligament
AU: Arbitraty unit
CMS: Constant murley score
DVT: Deep venous trombosis
DAP: Dose area product
HHS: Harris hip score
HTO: High tibial osteotomy
MDR: Medical device regulation
NA: Not applicable
NR: Not reported
NaN: Not a number
OSS: Oxford shoulder score
PSG: Patient specific guide
PROMS: Patient reported outcome measures
RCT: Randomized controlled trial
VAS: Visual analogue score
THA: Total hip arthroplasty
TSA: Total shoulder arthroplasty
