# Supplementary material for: Clinical added value of 3D printed patient-specific guides in orthopedic surgery (excluding knee arthroplasty): a systematic review
Source: Arch Orthop Trauma Surg. 2025 Mar 3;145(1):173. doi: 10.1007/s00402-025-05775-2 (PMC11872977; doi:10.1007/s00402-025-05775-2)
Supplement: Supplementary file 1 — (DOCX 23 KB) [file 402_2025_5775_MOESM1_ESM.docx]

**Supplementary data I**

**Literature Search**

Comple search performed on September 5^th^ 2023 in PubMed and Embase.

**Pubmed search (2952 hits):**

(

Orthopedic procedures [mesh] OR orthopedics [mesh] OR orthopedic* [tiab] OR orthopaedic* [tiab]

AND

templat* [tiab] OR guide [tiab] OR guides [tiab] OR instrument* [tiab] OR jigs [tiab] OR jig [tiab]

AND

Patient-specific modeling [mesh] OR Precision medicine/instrumentation [mesh] Printing, three-dimensional [mesh] OR Patient-specific [tiab] OR Custom* [tiab] OR Patient matched [tiab] OR Individual* [tiab] OR Personal* [tiab] OR Plan* [tiab] OR 3d [tiab] OR Threedimension* [tiab] OR three dimension* [tiab] OR 3 dimension* [tiab] OR osteotom* [tiab] OR cutting [tiab] OR drill* [tiab] OR saw [tiab] OR sawing [tiab] OR correction* [tiab]

AND

(randomized controlled trial[pt] OR controlled clinical trial[pt] OR randomized controlled trials[mh] OR random allocation[mh] OR double-blind method[mh] OR single-blind method[mh] OR clinical trial[pt] OR clinical trials[mh] OR "clinical trial"[tw] OR ((singl*[tw] OR doubl*[tw] OR trebl*[tw] OR tripl*[tw]) AND (mask*[tw] OR blind*[tw])) OR "latin square"[tw] OR placebos[mh] OR placebo*[tw] OR random*[tw] OR research design[mh:noexp] OR comparative study[pt] OR evaluation studies[pt] OR follow-up studies[mh] OR prospective studies[mh] OR cross-over studies[mh] OR control[tw] OR controll*[tw] OR prospectiv*[tw] OR volunteer*[tw]) NOT (animals[mh] NOT humans[mh])

NOT

Arthroplasty, Replacement, Knee [Mesh] OR knee replacement [ti] OR TKA [Ti] OR total knee arthroplasty* [Ti] OR Knee Arthroplasty [Ti] OR Unicompartmental Knee Arthroplasty* [Ti] OR UKA [ti]

) Filters: from 2008 – 2023

**Embase search (4622 hits)**

(

('orthopedic surgery'/exp OR orthopedic*:ti,ab,kw OR orthopaedic*:ti,ab,kw)

AND

(templat*:ti,ab,kw OR guide:ti,ab,kw OR guides:ti,ab,kw OR instrument*:ti,ab,kw OR jigs:ti,ab,kw OR jigs:ti,ab,kw)

AND

('personalized medicine'/exp OR 'guiding device'/exp OR 'three dimensional printing'/exp OR (((('patient specific':ti,ab,kw OR custom*:ti,ab,kw OR patient:ti,ab,kw) AND matched:ti,ab,kw OR individual*:ti,ab,kw OR personal*:ti,ab,kw OR plan*:ti,ab,kw OR 3d:ti,ab,kw OR threedimension*:ti,ab,kw OR three:ti,ab,kw) AND dimension*:ti,ab,kw OR 3:ti,ab,kw) AND dimension*:ti,ab,kw) OR osteotom*:ti,ab,kw OR cutting:ti,ab,kw OR drill*:ti,ab,kw OR saw:ti,ab,kw OR sawing:ti,ab,kw OR correction*:ti,ab,kw)

AND

('clinical trial'/exp OR 'triple blind procedure'/exp OR 'double blind procedure'/exp OR 'single blind procedure'/exp OR 'randomization'/exp OR 'placebo'/exp OR 'methodology'/de OR 'comparative study'/de OR 'evaluation study'/de OR 'follow up'/exp OR 'prospective study'/exp OR 'crossover procedure'/exp OR 'clinical trial':ab,ti OR ((singl*:ab,ti OR doubl*:ab,ti OR trebl*:ab,ti OR tripl*:ab,ti) AND (mask*:ab,ti OR blind*:ab,ti)) OR 'latin square':ab,ti OR placebo*:ab,ti OR random*:ab,ti OR control:ab,ti OR controll*:ab,ti OR prospectiv*:ab,ti OR volunteer*:ab,ti)

NOT

('knee replacement' OR tka OR 'total knee arthroplasty*' OR 'knee arthroplasty' OR 'unicompartmental knee arthroplasty*':ti,kw))

AND

(2008:py OR 2009: py OR 2010:py OR 2011:py OR 2012:py OR 2013:py OR 2014:py OR 2015:py OR 2016:py OR 2017:py OR 2018:py OR 2019:py OR 2020:py OR 2021:py OR 2022:py OR 2023:py)

)
