# Supplementary material for: Clinical added value of 3D printed patient-specific guides in orthopedic surgery (excluding knee arthroplasty): a systematic review
Source: Arch Orthop Trauma Surg. 2025 Mar 3;145(1):173. doi: 10.1007/s00402-025-05775-2 (PMC11872977; doi:10.1007/s00402-025-05775-2)
Supplement: Supplementary file 2 — (DOCX 298 KB) [file 402_2025_5775_MOESM2_ESM.docx]

**Supplementary data II**

| Study outcomes | | | | | | | | | | | | | | | | | |
| --- | --- | --- | --- | --- | --- | --- | --- | --- | --- | --- | --- | --- | --- | --- | --- | --- | --- |
|  | | | | | | | | | | | | | | | | | |
| 1. Patient reported outcome measures (PROMs) | | | | | | | | | | | | | | | | | |
| Application | Reference | N | Overall Risk of Bias | Patient outcome description | | Value in intervention group | | Value in control group | | P-value | | Absolute change (Δ) | | Relative change (Δ%) | | | Positive or negative  significant outcome for PSG |
| Spinal fusion | Zhang (2020)^52^ | 40 | ? | VAS pre-op | | 6.5 ± 1.1 | | 6.2 ± 1.2 | | 0.615 | | ~ | | ~ | | | ~ |
|  |  |  |  | VAS 1d post-op | | 3.2 ± 0.8 | | 6.5 ± 0.6 | | <0.01 | | -3.3 | | -51.2 | | | + |
|  |  |  |  | VAS 7d post-op | | 2.7 ± 0.8 | | 4.5 ± 1.1 | | 0.041 | | -1.8 | | -40.7 | | | + |
|  |  |  |  | VAS 1m post-op | | 1.8 ± 0.8 | | 2.0 ± 0.6 | | 0.687 | | -0.2 | | -8.5 | | | (+) |
|  |  |  |  | VAS 3m post-op | | 1.3 ± 0.5 | | 1.5 ± 0.6 | | 0.6 | | -0.2 | | -11.3 | | | (+) |
|  |  |  |  | ODI pre-op | | 81.2 ± 1.5 | | 82.2 ± 1.6 | | 0.287 | | ~ | | ~ | | | ~ |
|  |  |  |  | ODI 1d post-op | | 36.5 ± 1.1 | | 43.7 ± 2.0 | | <0.01 | | -7.2 | | -16.4 | | | + |
|  |  |  |  | ODI 7d post-op | | 27.2 ± 1.2 | | 35.5 ± 1.1 | | <0.01 | | -8.3 | | -23.5 | | | + |
|  |  |  |  | ODI 1m post-op | | 25.3 ± 0.8 | | 26.8 ± 0.4 | | 0.651 | | -1.5 | | -5.6 | | | (+) |
|  |  |  |  | ODI 3m post-op | | 14.2 ± 0.8 | | 16.2 ± 1.0 | | 0.627 | | -2.0 | | -12.4 | | | (+) |
|  | Merc (2017)^53^ | 24 | ? | VAS back pain pre-op | | 7.4 ± 1.4 | | 7.2 ± 1.2 | | 0.809 | | ~ | | ~ | | | ~ |
|  |  |  |  | VAS back pain 1m post-op | | 4.5 ± 2.2 | | 5.1 ± 1.6 | | 0.477 | | -0.6 | | -11.8 | | | (+) |
|  |  |  |  | VAS back pain >3y post-op | | 4.7 ± 2.1 | | 4.9 ± 2.6 | | 0.826 | | -0.2 | | -4.1 | | | (+) |
|  |  |  |  | VAS leg pain pre-op | | 7.2 ± 1.8 | | 7.5 ± 1.6 | | 0.612 | | ~ | | ~ | | | ~ |
|  |  |  |  | VAS leg pain 1m post-op | | 4.2 ± 1.7 | | 5.2 ± 1.5 | | 0.134 | | -1.0 | | -19.2 | | | (+) |
|  |  |  |  | VAS leg pain >3y post-op | | 4.1 ± 2.4 | | 4.5 ± 2.2 | | 0.651 | | -0.4 | | -8.9 | | | (+) |
|  | Feng (2012)^54^ | 12 | ? | Pre-op JOA score | | 8.7 ± 1.6 | | 7.7 ± 2.0 | | 0.36 | | ~ | | ~ | | | ~ |
|  |  |  |  | Post-op JOA score | | 12.8 ± 1.2 | | 11.8 ± 1.0 | | 0.14 | | 1.0 | | 8.5 | | | (+) |
|  |  |  |  | Improvement rate JOA score (%) | | 104.2 ± 23.4 | | 82.8 ± 29.9 | | 0.212 | | 21.4 | | 13.0 | | | (+) |
|  | Cui (2021)^55^ | 84 | - | Pre-op ODI | | 44.7 ± 10.5 | | 44.4 ± 10.3 | | >0.05 | | ~ | | ~ | | | ~ |
|  |  |  |  | 2y post-op ODI | | 19.2 ± 7.9 | | 20.5 ± 8.6 | | >0.05 | | -1.3 | | -6.4 | | | (+) |
|  |  |  |  | Pre-op SF-36 | | 83.1 ± 19.9 | | 82.6 ± 20.5 | | >0.05 | | ~ | | ~ | | | ~ |
|  |  |  |  | 2y post-op SF-36 | | 135.8 ± 32.5 | | 133.9 ± 30.1 | | >0.05 | | 1.9 | | 1.4 | | | (+) |
| Total hip arthroplasty | Wang (2021)^57^ | 104 | + | Divided into four subgroups based Crowe’s classification for hip dysplasia (Crow I, II, III, IV) | | | | | | | | | | | | |  |
|  |  | I |  | HHS pre-op | | 56.5 ± 10.4 | | 55.8 ± 13.3 | | 0.847 | | ~ | | ~ | | | ~ |
|  |  |  |  | HHS 3m post-op | | 85.2 ± 3.6 | | 84.5 ± 3.3 | | 0.499 | | 0.7 | | 0.9 | | | (+) |
|  |  |  |  | HHS 1y post-op | | 88.4 ± 4.7 | | 90.5 ± 3.8 | | 0.088 | | -2.0 | | -2.2 | | | (-) |
|  |  | II |  | HHS pre-op | | 52.9 ± 13.1 | | 54.3 ± 9.0 | | 0.757 | | ~ | | ~ | | | ~ |
|  |  |  |  | HHS 3m post-op | | 85.9 ± 4.4 | | 84.3 ± 6.3 | | 0.458 | | 1.6 | | 1.9 | | | (+) |
|  |  |  |  | HHS 1y post-op | | 89.8 ± 5.3 | | 87.2 ± 3.3 | | 0.170 | | 2.5 | | 2.9 | | | (+) |
|  |  | III |  | HHS pre-op | | 44.9 ± 17.1 | | 43.0 ± 16.9 | | 0.835 | | ~ | | ~ | | | ~ |
|  |  |  |  | HHS 3m post-op | | 83.1 ± 5.1 | | 77.7 ± 4.3 | | 0.043 | | 5.4 | | 7.0 | | | + |
|  |  |  |  | HHS 1y post-op | | 88.9 ± 5.5 | | 82.4 ± 2.9 | | 0.015 | | 6.4 | | 7.8 | | | + |
|  |  | IV |  | HHS pre-op | | 40.3 ± 13.8 | | 41.0 ± 11.2 | | 0.902 | | ~ | | ~ | | | ~ |
|  |  |  |  | HHS 3m post-op | | 71.9 ± 6.7 | | 70.1 ± 7.1 | | 0.566 | | 1.8 | | 2.6 | | | (+) |
|  |  |  |  | HHS 1y post-op | | 87.3 ± 3.5 | | 83.2 ± 4.3 | | 0.031 | | 4.1 | | 4.9 | | | + |
|  | Jin (2022)^58^ | 80 | + | VAS pre-op | | 6.4 ± 0.9 | | 6.3 ± 1.1 | | 0.657 | | ~ | | ~ | | | ~ |
|  |  |  |  | VAS 7d post-op | | 2.2 ± 0.6 | | 2.3 ± 0.8 | | 0.42 | | -0.1 | | -5.6 | | | (+) |
|  |  |  |  | VAS 4w post-op | | 1.6 ± 0.5 | | 1.7 ± 0.8 | | 0.41 | | -0.1 | | -7.5 | | | (+) |
|  |  |  |  | VAS 3m post-op | | 1.4 ± 0.7 | | 1.3 ± 0.6 | | 0.857 | | 0.1 | | +1.5 | | | (-) |
|  |  |  |  | VAS 6m post-op | | 0.8 ± 0.6 | | 0.9 ± 0.6 | | 0.713 | | -0.1 | | -10.8 | | | (+) |
|  |  |  |  | VAS 12m post-op | | 0.7 ± 0.5 | | 0.7 ± 0.6 | | 0.845 | | 0.0 | | 0.0 | | | = |
|  |  |  |  | HHS pre-op | | 48.9 ± 4.8 | | 49.7 ± 5.6 | | 0.536 | | ~ | | ~ | | | ~ |
|  |  |  |  | HHS 7d post-op | | 82.8 ± 5.0 | | 76.8 ± 6.4 | | <0.001 | | 6.0 | | 7.7 | | | + |
|  |  |  |  | HHS 4w post-op | | 89.2 ± 2.8 | | 85.9 ± 3.1 | | <0.001 | | 3.3 | | 3.8 | | | + |
|  |  |  |  | HHS 3m post-op | | 93.9 ± 1.7 | | 92.7 ± 1.8 | | 0.003 | | 1.2 | | 1.3 | | | + |
|  |  |  |  | HHS 6m post-op | | 95.0 ± 1.8 | | 94.5 ± 2.2 | | 0.156 | | 0.5 | | 0.5 | | | (+) |
| Anterior cruciate ligament reconstruction | Zhu (2018)^63^ | 78 | ? | Lysholm score pre-op | | 59.4 ± 15.1 | | 63.1 ± 14.1 | | 0.185 | | ~ | | ~ | | | ~ |
|  |  |  |  | Lysholm score follow-up | | 89.3 ± 7.6 | | 87.4 ± 10.0 | | 0.258 | | 1.9 | | 2.2 | | | (+) |
|  |  |  |  | IKDC score pre-op | | 56.7 ± 11.9 | | 60.2 ± 15.2 | | 0.392 | | ~ | | ~ | | | ~ |
|  |  |  |  | IKDC score follow-up | | 80.9 ± 10.5 | | 79.7 ± 9.6 | | 0.751 | | 1.2 | | 1.5 | | | (+) |
|  | Liu (2020)^64^ | 41 | ? | Lysholm score pre-op | | 46.7 ± 4.3 | | 46.9 ± 4.1 | | 0.871 | | ~ | | ~ | | | ~ |
|  |  |  |  | Lysholm score 6m post-op | | 81.2 ± 4.2 | | 80.2 ± 5.4 | | 0.347 | | 1.0 | | 1.2 | | | (+) |
|  |  |  |  | Lysholm score 12m post-op | | 82.0 ± 4.4 | | 81.3 ± 5.3 | | 0.303 | | 0.7 | | 0.9 | | | (+) |
|  |  |  |  | IKDC score pre-op | | 45.9 ± 5.2 | | 45.6 ± 5.8 | | 0.868 | | ~ | | ~ | | | ~ |
|  |  |  |  | IKDC score 6m post-op | | 83.9 ± 3.2 | | 82.9 ± 3.5 | | 0.339 | | 1.0 | | 1.2 | | | (+) |
|  |  |  |  | IKDC score 12m post-op | | 87.2 ± 5.6 | | 85.7 ± 5.6 | | 0.380 | | 1.6 | | 1.8 | | | (+) |
|  | Lan (2023)^65^ | 80 | - | HHS 12m post-op | | 95.3 ± 1.1 | | 94.9 ± 1.7 | | 0.189 | | 0.4 | | 0.4 | | | (+) |
|  |  |  |  | Lysholm score 3m post-op | | 93.2 ± 1.2 | | 93.1 ± 1.2 | | >0.05 | | 0.1 | | 0.1 | | | (+) |
|  |  |  |  | Lysholm score 6m post-op | | 93.8 ± 1.2 | | 93.8 ± 1.1 | | >0.05 | | 0.1 | | 0.1 | | | (+) |
|  |  |  |  | Lysholm score 12 m post-op | | 94.6 ± 0.8 | | 94.9 ± 1.0 | | >0.05 | | -0.3 | | -0.3 | | | (-) |
|  |  |  |  | IKDC score pre-op | | 44.4 ± 3.5 | | 45.0 ± 3.4 | | >0.05 | | ~ | | ~ | | | ~ |
|  |  |  |  | IKDC score 3m post-op | | 93.8 ± 1.3 | | 93.9 ± 1.2 | | >0.05 | | -0.1 | | -0.1 | | | (-) |
|  |  |  |  | IKDC score 6m post-op | | 94.2 ± 1.1 | | 94.0 ± 1.3 | | >0.05 | | 0.1 | | 0.1 | | | (+) |
|  |  |  |  | IKDC score 12 m post-op | | 95.0 ± 1.0 | | 94.9 ± 1.1 | | >0.05 | | 0.2 | | 0.2 | | | (+) |
| High tibial osteotomy | Gao (2021)^66^ | 39 | + | Clinical AKSS pre-op | | 57.5 ± 2.0 | | 57.1 ± 2.4 | | 0.616 | | ~ | | ~ | | | ~ |
|  |  |  |  | Clinical AKSS 3m post-op | | 75.6 ± 7.3 | | 70.2 ± 8.4 | | 0.042 | | 5.5 | | 7.8 | | | + |
|  |  |  |  | Clinical AKSS 6m post-op | | 85.0 ± 6.1 | | 80.2 ± 7.4 | | 0.040 | | 4.8 | | 6.0 | | | + |
|  |  |  |  | Clinical AKSS last follow-up | | 90.3 ± 6.2 | | 89.4 ± 6.3 | | 0.654 | | 0.9 | | 1.0 | | | (+) |
|  |  |  |  | Functional AKSS pre-op | | 58.8 ± 6.2 | | 59.1 ± 7.6 | | 0.870 | | ~ | | ~ | | | ~ |
|  |  |  |  | Functional AKSS 3m post-op | | 70.9 ± 8.0 | | 64.8 ± 9.0 | | 0.034 | | 6.2 | | 9.5 | | | + |
|  |  |  |  | Functional AKSS 6m post-op | | 81.6 ± 4.7 | | 80.4 ± 8.3 | | 0.625 | | 1.1 | | 1.4 | | | (+) |
|  |  |  |  | Functional AKSS last follow-up | | 90.0 ± 6.3 | | 89.6 ± 6.4 | | 0.835 | | 0.4 | | 0.5 | | | (+) |
|  | Zhu (2022)^67^ | 96 | ? | VAS pre-op – scale [0-100] | | 46.0 ± 5.7 | | 45.4 ± 6.9 | | NR | | ~ | | ~ | | | ~ |
|  |  |  |  | VAS 1m post-op – scale [0-100] | | 30.8 ± 6.2 | | 35.8 ± 6.2 | | 0.016 | | -5.0 | | -14.0 | | | + |
|  |  |  |  | VAS 6m post-op – scale [0-100] | | 19.9 ± 5.5 | | 22.4 ± 6.5 | | 0.006 | | -2.5 | | -11.2 | | | + |
|  |  |  |  | VAS 12m post-op – scale [0-100] | | 15.1 ± 4.3 | | 19.1 ± 4.4 | | <0.001 | | -4.0 | | -20.9 | | | + |
|  |  |  |  | VAS last follow-up – scale [0-100] | | 18.2 ± 4.4 | | 20.5 ± 4.8 | | 0.031 | | -2.3 | | -11.2 | | | + |
|  |  |  |  | Lysholm score pre-op | | 56.3 ± 14.1 | | 56.4 ± 11.3 | | NR | | ~ | | ~ | | | ~ |
|  |  |  |  | Lysholm score 1m post-op | | 73.7 ± 10.0 | | 64.4 ± 10.8 | | 0.027 | | 9.3 | | 14.4 | | | + |
|  |  |  |  | Lysholm score 6m post-op | | 85.9 ± 9.9 | | 76.2 ± 11.5 | | 0.007 | | 9.7 | | 12.7 | | | + |
|  |  |  |  | Lysholm score 12m post-op | | 82.2 ± 9.1 | | 69.6 ± 10.5 | | 0.002 | | 12.6 | | 18.1 | | | + |
|  |  |  |  | Lysholm score last follow-up | | 74.8 ± 9.1 | | 66.7 ± 10.9 | | 0.045 | | 8.1 | | 12.1 | | | + |
|  |  |  |  | WOMAC score pre-op | | 105.7 ± 17.1 | | 105.5 ± 15.8 | | NR | | ~ | | ~ | | | ~ |
|  |  |  |  | WOMAC score 1m post-op | | 81.1 ± 11.0 | | 94.8 ± 12.4 | | 0.020 | | -13.7 | | -14.5 | | | + |
|  |  |  |  | WOMAC score 6m post-op | | 70.9 ± 11.1 | | 82.7 ± 11.9 | | 0.008 | | -11.8 | | -14.3 | | | + |
|  |  |  |  | WOMAC score 12m post-op | | 74.6 ± 10.6 | | 87.1 ± 12.3 | | 0.004 | | -12.5 | | -14.4 | | | + |
|  |  |  |  | WOMAC score last follow-up | | 89.1 ± 10.8 | | 89.2 ± 11.5 | | NR | | -0.1 | | -0.1 | | | (+) |
| Total shoulder arthroplasty | Boekel (2023)^68^ † | 47 | + | CMS pre-op | | 30.2 ± 17.6 | | 26.6 ± 16.3 | | 0.48 | | ~ | | ~ | | | ~ |
|  |  |  |  | CMS 6m post-op | | 68.6 ± 13.8 | | 60.3 ± 16.3 | | 0.06 | | 8.3 | | 13.8 | | | (+) |
|  |  |  |  | ASES score pre-op | | 33.7 ± 15.1 | | 35.4 ± 10.5 | | 0.64 | | ~ | | ~ | | | ~ |
|  |  |  |  | ASES score 6m post-op | | 78.1 ± 16.0 | | 75.3 ± 18.8 | | 0.58 | | 2.8 | | 3.7 | | | (+) |
|  |  |  |  | OSS pre-op | | 24.3 ± 7.5 | | 23.1 ± 6.1 | | 0.57 | | ~ | | ~ | | | ~ |
|  |  |  |  | OSS 6m post-op | | 40.7 ± 5.7 | | 38.4 ± 6.6 | | 0.20 | | 2.3 | | 6.1 | | | (-) |
| Percutaneous vertebroplasty | Hu (2021)^70^ | 36 | ? | VAS pre-op | | 8.4 ± 0.5 | | 8.4 ± 0.5 | | >0.05 | | ~ | | ~ | | | ~ |
|  |  |  |  | VAS post-op | | 2.4 ± 0.6 | | 2.3 ± 0.7 | | >0.05 | | 0.1 | | 4.3 | | | (-) |
|  | Chen (2022)^71^ | 97 | - | VAS pre-op | | 8.2 ± 1.1 | | 7.8 ± 1.4 | | 0.146 | | ~ | | ~ | | | ~ |
|  |  |  |  | VAS 7d post-op | | 3.8 ± 1.4 | | 4.5 ±1.6 | | 0.016 | | -0.7 | | -0.2 | | | + |
|  |  |  |  | VAS 3m post-op | | 1.5 ± 0.5 | | 1.5 ± 0.8 | | 0.954 | | 0.0 | | 0.0 | | | = |
|  |  |  |  | ODI pre-op | | 41.5 ± 4.3 | | 39.8 ± 5.0 | | 0.079 | | ~ | | ~ | | | ~ |
|  |  |  |  | ODI 7d post-op | | 25.2 ± 6.2 | | 30.6 ± 5.8 | | <0.001 | | -5.4 | | -17.5 | | | + |
|  |  |  |  | ODI 3m post-op | | 16.9 ± 6.3 | | 22.3 ± 5.7 | | <0.001 | | -5.4 | | -24.3 | | | + |
| Distal radius osteotomy | Buijze (2018)^72^ | 40 | ? | Pain score pre-op | | 6.2 ± 2.5 | | 5.9 ± 2.8 | | 0.916 | | ~ | | ~ | | | ~ |
|  |  |  |  | Pain score 3m post-op | | 3.4 ± 2.3 | | 2.4 ± 2.2 | | 0.343 | | 1.0 | | 41.7 | | | (-) |
|  |  |  |  | Pain score 6m post-op | | 3.2 ± 2.4 | | 2.6 ± 2.8 | | 0.985 | | 0.6 | | 23.1 | | | (-) |
|  |  |  |  | Pain score 12m post-op | | 2.8 ± 2.1 | | 2.1 ± 1.8 | | 0.903 | | 0.7 | | 33.3 | | | (-) |
|  |  |  |  | DASH score pre-op | | 51.8 ± 20.6 | | 46.2 ± 25.0 | | 0.598 | | ~ | | ~ | | | ~ |
|  |  |  |  | DASH score 3m post-op | | 33.8 ± 24.0 | | 27.9 ± 19.0 | | 0.546 | | 5.9 | | 21.1 | | | (-) |
|  |  |  |  | DASH score 6m post-op | | 24.1 ± 20.1 | | 23.8 ± 18.9 | | 0.299 | | 0.3 | | 1.3 | | | (-) |
|  |  |  |  | DASH score 12m post-op | | 18.8 ± 18.0 | | 22.8 ±20.4 | | 0.103 | | -4.0 | | -17.5 | | | (+) |
|  |  |  |  | PRWE score pre-op | | 58.2 ± 17.2 | | 50.4 ± 20.1 | | 0.217 | | ~ | | ~ | | | ~ |
|  |  |  |  | PRWE score 3m post-op | | 35.3 ± 28.7 | | 28.7 ± 23.4 | | 0.790 | | 6.6 | | 23.0 | | | (-) |
|  |  |  |  | PRWE score 6m post-op | | 22.8 ± 15.0 | | 22.8 ± 16.7 | | 0.283 | | 0.0 | | 0.0 | | | = |
|  |  |  |  | PRWE score 12m post-op | | 25.3 ± 20.6 | | 22.3 ± 24.3 | | 0.226 | | 3.0 | | 13.5 | | | (-) |
|  |  |  |  | Satisfaction score pre-op | | 2.8 ± 2.4 | | 3.0 ± 1.7 | | 0.8 | | ~ | | ~ | | | ~ |
|  |  |  |  | Satisfaction score 3m post-op | | 7.5 ± 2.0 | | 8.4 ± 1.4 | | 0.916 | | -0.9 | | -10.7 | | | (-) |
|  |  |  |  | Satisfaction score 6m post-op | | 7.6 ± 1.8 | | 7.7 ± 2.2 | | 0.542 | | -0.1 | | -1.3 | | | (-) |
|  |  |  |  | Satisfaction score 12m post-op | | 8.0 ± 1.9 | | 8.4 ± 1.7 | | 0.640 | | -0.4 | | -4.8 | | | (-) |
| Distal humerus osteotomy | Hu (2020)^73^ | 35 | ? | Satisfied with the result (n, [%]) | | 16 (100) | | 16 (84.2) | | 0.234 | | 15.8 | | 18.8 | | | (+) |
| Acetabular osteotomy | Ma (2022)^75^ | 22 | - | HHS pre-op | | 66.0 ± 0.7 | | 67.5 ± 0.6 | | >0.05 | | ~ | | ~ | | | ~ |
|  |  |  |  | HHS 6m post-op | | 88.8 ± 0.5 | | 87.6 ± 0.6 | | >0.05 | | 1.3 | | 1.5 | | | (+) |
|  |  |  |  | Improvement HHS | | 21.9 ± 0.9 | | 20.1 ± 0.6 | | >0.05 | | 1.8 | | 9.1 | | | (+) |
| Femoral neck fracture repair | Wang (2021)^76^ | 60 | - | HHS post-op 12m | | 93.7 ± 4.0 | | 91.6 ± 4.2 | | 0.052 | | 2.1 | | 2.3 | | | (+) |
|  |  |  | |  | |  | |  | |  | |  | | | | |  |
|  |  |  | |  | |  | |  | |  | |  | | | | |  |
| 1. Complications | | | | | | | | | | | | | | | | | |
| Application | Reference | N | Overall Risk of Bias | Complication type | Number in intervention group | | Number in control group | | P-value | | Absolute change (Δ) | | Relative change (Δ%) | | | Positive or negative  significant outcome for PSG | |
| Spinal fusion | Cecchinato (2019)^50^ | 29 | + | Malpositioned implants, Grade B – C (n, [%]) | 29 (9.8) | | 41 (16.9) | | 0.014 | | -7.1 | | -42.0 | | | + | |
|  |  |  |  | Malpositioned implants, Grade C (n, [%]) | 7 (2.4) | | 22 (9.1) | | <0.001 | | -6.7 | | -73.6 | | | + | |
|  |  |  |  | Dural lesion (n, [%]) | 1 (7.1) | | 1 (6.7) | | NR | | 0.5 | | 7.1 | | | (-) | |
|  | Chen (2015)^51^ | 43 | ? | Malpositioned implants (n, [%]) | 0 (0) | | 2 (1.6) | | NR | | -1.6 | | -100 | | | (+) | |
|  | Zhang (2020)^52^ | 40 | ? | Vertebral pedicles damaged (n, [%]) | 1 (5) | | 8 (40) | | 0.029 | | -35 | | -87.5 | | | + | |
|  | Merc (2017)^53^ | 24 | ? | Cortex perforations (n, [%]) | 6 (8.3) | | 29 (40.2) | | <0.01 | | -31.9 | | -79.3 | | | + | |
|  |  |  |  | Laminectomy (n, [%]) | 1 (1.4) | | 0 (0) | | NR | | 1.4 | | NaN | | | (-) | |
|  |  |  |  | Osteoporotic vertebral fracture (n, [%]) | 0 (0) | | 1 (1.4) | | NR | | -1.4 | | -100 | | | (+) | |
|  | Feng (2012)^54^ | 12 | ? | No complications occurred in both groups | | | | | | | | | | | | | |
|  | Cui (2021)^55^ | 84 | - | Incision infection (n, [%]) | 1 (2.2) | | 3 (7.1) | | NR | | -4.9 | | -68.8 | | | (+) | |
|  |  |  |  | Screw exposure (n, [%]) | 0 (0) | | 1 (2.4) | | NR | | -2.4 | | -100 | | | (+) | |
|  |  |  |  | Screw loosening and dropping (n, [%]) | 2 (4.8) | | 4 (9.5) | | NR | | -4.8 | | -50.0 | | | (+) | |
|  |  |  |  | Pseudo articulation formation (n, [%]) | 1 (2.4) | | 2 (4.8) | | NR | | -2.4 | | -50.0 | | | (+) | |
|  |  |  |  | Rod breaking (n, [%]) | 1 (2.4) | | 1 (2.4) | | NR | | 0 | | 0 | | | = | |
|  |  |  |  | Total (n, [%]) | 5 (11.9) | | 11 (26.2) | | 0.012 | | -14.3 | | -54.5 | | | + | |
|  | Merc (2013)^56^ | 19 | - | Cortex perforations (n, [%]) | 6 (11.1) | | 21 (38.9) | | <0.001 | | -27.8 | | -71.4 | | | + | |
|  |  |  |  | Screw length violations (n, [%]) | 14 (25.9) | | 20 (37.0) | | 0.21 | | -11.1 | | -30.0 | | | (+) | |
| Total hip arthroplasty | Wang (2021)^57^ | 104 | + | Divided into four subgroups based Crowe’s classification for hip dysplasia (Crowe I, II, III, IV) | | | | | | | | | | | | | |
|  |  | III |  | DVT | 1 (12.5) | | 0 (0) | | NR | | 12.5 | | NaN | | | (-) | |
|  |  | IV |  | DVT | 0 (0) | | 1 (10) | | NR | | -10 | | -100 | | | (+) | |
|  |  |  |  | Transient paralysis of peroneal nerve (n, [%]) | 0 (0) | | 1 (10) | | NR | | -10 | | -100 | | | (+) | |
|  |  |  |  | Transient paralysis of femoral nerve (n, [%]) | 0 (0) | | 1 (10) | | NR | | -10 | | -100 | | | (+) | |
|  | Jin (2022)^58^ | 80 | + | No complications occurred in both groups | | | | | | | | | | | | | |
|  | Small (2014)^60^ | 36 | - | Postoperative anterior dislocation (n, [%]) | 0 (0) | | 1 (5.6) | | NR | | -5.6 | | -100 | | | (+) | |
| Anterior cruciate ligament reconstruction | Zhu (2018)^63^ | 78 | ? | No complications occurred in both groups | | | | | | | | | | | | | |
|  | Liu (2020)^64^ | 41 | ? | Hypoesthesia of the infrapatellar nerve (n, [%]) | 2 (9.1) | | 1 (5.3) | | NR | | 3.8 | | 72.7 | | | (-) | |
|  | Lan (2023)^65^ | 80 | - | No complications occurred in both groups | | | | | | | | | | | | | |
| High tibial osteotomy | Gao (2021)^66^ | 39 | + | Lateral hinge fracture (n, [%]) | 0 (0) | | 2 (8.7) | | NR | | -8.7 | | -100 | | | (+) | |
|  |  |  |  | Incisional exudation (n, [%]) | 1 (6.3) | | 0 (0) | | NR | | 6.3 | | NaN | | | (-) | |
|  | Zhu (2022)^67^ | 96 | ? | Early superficial surgical wound infection (n, [%]) | 1 (2.1) | | 2 (4.2) | | NR | | -2.1 | | -50 | | | (+) | |
|  |  |  |  | Lateral hinge fracture (n, [%]) | 1 (2.1) | | 1 (2.1) | | NR | | 0 | | 0 | | | = | |
|  |  |  |  | Concomitant cartilage lesion (n, [%]) | 2 (4.2) | | 2 (4.2) | | NR | | 0 | | 0 | | | = | |
|  |  |  |  | Number of complications (n, [%]) | 4 (10.5) | | 5 (12.5) | | 0.416 | | -2.1 | | -20.0 | | | (+) | |
| Total shoulder arthroplasty | Boekel (2023)^68^ † | 47 | + | Anterior dislocation (n, [%]) | 1 (4.1) | | 0 (0) | | NR | | 4.1 | | NaN | | | (-) | |
|  |  |  |  | Subacromial bursitis (n, [%]) | 0 (0) | | 1 (4.3) | | NR | | -4.3 | | -100 | | | (+) | |
|  |  |  |  | Sirveaux grade I + II scapular notching (n, [%]) | 2 (8.3) | | 3 (13.0) | | 0.73 | | -4.7 | | -36.1 | | | (+) | |
|  | Hendel (2020)^69^ | 31 | + | Nonoptimal implant type used during surgery (n [%]) | 1 (7) | | 10 (63) | | <0.001 | | -56 | | -88.9 | | | + | |
|  |  |  |  | Version or inclination malposition occurrences (n [%]) | 4 (13) | | 14 (44) | | <0.001 | | -31 | | -221.4 | | | + | |
|  |  |  |  | Malpositioned implants with >10° deviation in version and/or inclination from the optimal preoperative plan (n [%]) | 4 (27) | | 12 (75) | | <0.01 | | -48.0 | | -400 | | | + | |
|  |  |  |  | Transient partial axillary nerve injury (n [%]) | 0 (0) | | 1 (5.9) | | - | | -5.9 | | NaN | | | (+) | |
| Percutaneous vertebroplasty | Hu (2021)^70^ | 36 | ? | Complications (n, [%]) | 3 (16.7) | | 7 (38.9) | | >0.05 | | -22.2 | | -57.1 | | | (+) | |
|  | Chen (2022)^71^ | 97 | - | Nerve injury (n, [%]) | 1 (2.1) | | 3 (6.0) | | - | | -3.9 | | -64.5 | | | (+) | |
|  |  |  |  | Vertebral collapse, peri-cement bone absorption (n, [%]) | 0 (0) | | 1 (2.0) | | - | | -2.0 | | -100 | | | (+) | |
|  |  |  |  | Cardiopulmonary complication (n, [%]) | 1 (2.1) | | 1 (2.0) | | - | | 0.1 | | 6.4 | | | (-) | |
|  |  |  |  | Bone-cement leakage (n, [%]) | 2 (4.3) | | 6 (12.0) | | - | | -7.7 | | -64.5 | | | (+) | |
| Distal radius osteotomy | Buijze (2018)^72^ | 40 | ? | Patients with complications (n, [%]) | 6 (30.0) | | 6 (35.3) | | - | | -5.3 | | -15 | | | (+) | |
|  |  |  |  | Total complications (n, [%]) | 7 (35.0) | | 7 (41.2) | | - | | -6.2 | | -15 | | | (+) | |
| Distal humerus osteotomy | Hu (2020)^73^ | 35 | ? | No complications occurred in both groups | | | | | | | | | | | | | |
| Correction of lower limb deformities | Fan (2022)^74^ | 55 | - | Growth plate cartilage or articular cartilage injury (n, [%]) | 0 (0) | | 3 (12.5) | | - | | -12.5 | | -100 | | | (+) | |
| Acetabular osteotomy | Ma (2022)^75^ | 22 | - | No complications occurred in both groups | | | | | | | | | | | | | |
| Femoral neck fracture repair | Wang (2021)^76^ | 60 | - | Femoral head necrosis (n, [%]) | 1 (3.3) | | 6 (20.0) | | 0.044 | | -16.7 | | -83.5 | | | + | |
|  | | | | | | | | | | | | | | | | | |
|  | | | | | | | | | | | | | | | | | |
| 1. Accuracy | | | | | | | | | | | | | | | | | |
| Application | Reference | N | Overall Risk of Bias | Method of measuring accuracy | Outcomes intervention group | | Outcomes control group | | P-value | | Absolute change (Δ) | | Relative change (Δ%) | | | Positive or negative  significant outcome for PSG | |
| Spinal fusion | Cecchinato (2019)^50^ | 29 | + | Implants placed in safe zone, grade 0-A (n [%])* | 268 (90.2) | | 202 (83.1) | | NR | | 7.1 | | 8.5 | | | (+) | |
|  | Zhang (2020)^52^ | 40 | ? | Pedicles successful pierced at first attempt (n, [%]) | 10 (12.5) | | 3 (3.8) | | 0.043 | | 8.8 | | 233 | | | + | |
|  |  |  |  | Insertions before reaching the desired position (n) | 7.8 ± 1.5 | | 17.5 ± 1.9 | | <0.01 | | -9.7 | | -55 | | | + | |
|  | Feng (2012)^54^ | 12 | ? | Excellent and good rate of screw positioning (n [%]) | 30 (83.3) | | 17 (47.2) | | 0.001 | | 36.1 | | 76.5 | | | + | |
|  |  |  |  | Bayard’s criterion for acceptable screw positioning (n [%]) | 32 (88.9) | | 22 (61.1) | | 0.014 | | 27.8 | | 45.5 | | | + | |
|  | Cui (2021)^55^ | 84 | - | Deviation screw positioning sagittal angle (°) | 0.04 ± 0.02 | | 0.19 ± 0.05 | | <0.001 | | -0.15 | | -78.9 | | | + | |
|  |  |  |  | Deviation screw positioning transverse angle (°) | 0.06 ± 0.02 | | 0.13 ± 0.04 | | <0.001 | | -0.07 | | -53.8 | | | + | |
|  |  |  |  | Horizontal distance from entry point to median sacral crest (mm) | 0.07 ± 0.04 | | 0.20 ± 0.07 | | <0.001 | | -0.13 | | -65.0 | | | + | |
|  |  |  |  | Vertical distance from the entry point to the superior margin of the second posterior sacral foramina (mm) | 0.02 ± 0.01 | | 0.15 ± 0.03 | | <0.001 | | -0.13 | | -86.7 | | | + | |
|  | Merc (2013)^56^ | 19 | - | Sagittal screw displacement (mm) | 0.3 ± 3.4 | | 1.5 ± 3.2 | | 0.05 | | -1.2 | | -80.0 | | | + | |
|  |  |  |  | Sagittal angular screw deviation (°) | -1 ± 5 | | -6 ± 8 | | <0.001 | | 5.0 | | 83.3 | | | + | |
|  |  |  |  | Transversal screw displacement (mm) | -0.7 ± 1.5 | | -0.2 ± 2.6 | | 0.28 | | -0.5 | | -250.0 | | | (+) | |
|  |  |  |  | Transversal angular screw deviation (°) | -1 ± 5 | | 0 ± 11 | | 0.71 | | 1.1 | | NaN | | | (+) | |
| Total hip arthroplasty | Jin (2022)^58^ | 80 | + | Absolute leg length discrepancy (mm) | 1.3 ± 1.0 | | 4.7 ± 2.6 | | <0.001 | | -3.5 | | -73.5 | | | + | |
|  |  |  |  | Leg length discrepancy ≤ 5 mm (n, [%]) | 40 (100) | | 28 (70) | | <0.001 | | 30 | | 42.9 | | | + | |
|  |  |  |  | Absolute femoral stem offset (mm) | 3.0 ± 1.6 | | 6.0 ± 2.7 | | <0.001 | | -3.0 | | -49.8 | | | + | |
|  |  |  |  | Absolute femoral stem offset ≤ 5 mm (n, [%]) | 36 (90) | | 27 (67.5) | | 0.014 | | 22.5 | | 33.3 | | | + | |
|  |  |  |  | Absolute femoral stem anteversion (°) | 3.5 ± 1.1 | | 6.9 ± 2.3 | | <0.001 | | -3.3 | | -48.5 | | | + | |
|  |  |  |  | Absolute femoral stem varus/valgus (°) | 0.8 ± 0.4 | | 2.4 ± 1.3 | | <0.001 | | -1.5 | | -64.4 | | | + | |
|  | Zhang (2011)^59^ | 20 | ? | Deviation of cup abduction angle (°) | 1.2 ± 0.9 | | 5.4 ± 3.2 | | <0.05 | | -4.2 | | -77.8 | | | + | |
|  |  |  |  | Deviation of cup anteversion angle (°) | 2.1 ± 1.2 | | 4.1 ± 2.8 | | <0.05 | | -2.0 | | -48.8 | | | + | |
|  |  |  |  | Deviation of the femoral short stem-shaft angle (°) | 1.3 ± 1.0 | | 10.2 ± 1.5 | | <0.05 | | -8.9 | | -73.5 | | | + | |
|  | Small (2014)^60^ | 36 | - | Difference in version (°) | -0.2 ± 6.9 | | -6.9 ± 8.9 | | 0.018 | | -6.7 | | -96.8 | | | + | |
|  |  |  |  | Difference in abduction angle (°) | -2.0 ± 7.3 | | 1.3 ± 9.1 | | 0.25 | | -3.3 | | -253 | | | (-) | |
|  | Zhang (2011)^61^ | 22 | - | Deviation between ideal abduction angle of 45° (°) | 1.6 ± 0.4 | | 5.8 ± 2.9 | | <0.05 | | -4.2 | | -72.4 | | | + | |
|  |  |  |  | Deviation between ideal anteversion angle of 18° (°) | 1.9 ± 1.1 | | 3.9 ± 2.5 | | <0.05 | | -2.0 | | -51.3 | | | + | |
|  | Zhang (2021)^62^ | 53 | - | Anteversion change (°) | 2.3 ± 1.9 | | 5.4 ± 3.7 | | <0.001 | | -3.1 | | -57.2 | | | + | |
|  |  |  |  | Cases with >5° difference (n, [%]) | 2 (8.7) | | 15 (50) | | 0.002 | | -41.3 | | -82.6 | | | + | |
| Anterior cruciate ligament reconstruction | Zhu (2018)^63^ | 78 | ? | Radiographic ideal tunnel (n, [%]) | 39 (97.5) | | 30 (78.9) | | 0.027 | | 18.6 | | 23.6 | | | + | |
|  |  |  |  | Center point deviation Dx (mm) | 1.0 ± 0.7 | | 1.9 ± 0.8 | | 0.001 | | -0.9 | | -46.8 | | | + | |
|  |  |  |  | Center point deviation Dy (mm) | 1.2 ± 0.54 | | 1.9 ± 0.8 | | 0.001 | | -0.7 | | -37.4 | | | + | |
|  |  |  |  | Center point deviation Dz (mm) | 0.9 ± 0.5 | | 1.4 ± 0.7 | | 0.001 | | 0.53 | | -37.3 | | | + | |
|  | Liu (2020)^64^ | 41 | ? | Percentage of the depth of the femoral tunnel insertion point (%) | 27.6 ± 4.2 | | 30.4 ± 3.7 | | 0.025 | | -2.8 | | -9.2 | | | + | |
|  |  |  |  | Percentage of the height of the femoral tunnel insertion point (%) | 36.0 ± 8.1 | | 40.7 ± 6.4 | | 0.045 | | -4.7 | | -11.5 | | | + | |
|  |  |  |  | Percentage of the sagittal plane of the tibial tunnel insertion point (%) | 38.1 ± 2.2 | | 36.5 ± 2.6 | | 0.038 | | 1.6 | | 4.38 | | | + | |
|  |  |  |  | Percentage of the coronal plane of the tibial tunnel insertion point (%) | 47.2 ± 1.9 | | 45.3 ± 2.5 | | 0.01 | | 1.9 | | 4.2 | | | + | |
|  | Lan (2023)^65^ | 80 | - | Lateral femoral positioning of reconstructed ACL femoral tunnel in relation to the medial side of the femur (AU) | 0.56 ± 0.02 | | 0.55 ± 0.02 | | >0.05 | | 0.01 | | 1.8 | | | ? | |
|  |  |  |  | Tunnel in relation to medial side of femur (mm)  Lateral femoral positioning of reconstructed ACL femoral tunnel in relation to intercondylar fossa of the femur (AU) | 0.32 ± 0.01 | | 0.32 ± 0.02 | | >0.05 | | 0.0 | | 0.0 | | | = | |
|  |  |  |  | Lateral femoral positioning of reconstructed ACL femoral tunnel in relation to intercondylar fossa of femur (°) | 47.97 ± 1.73 | | 48.09 ± 1.56 | | >0.05 | | -0.12 | | 0.48 | | | ? | |
|  |  |  |  | Angle formed by long axis of femoral stem and centerline of femoral tunnel (°) | 31.98 ± 2.29 | | 31.5 ± 2.24 | | >0.05 | | 0.48 | | 1.5 | | | ? | |
| High tibial osteotomy | Gao (2021)^66^ | 39 | + | Absolute difference from designed target | | | | | | | | | | | |  | |
|  |  |  |  | Weight-bearing line ratio (AU) | 2.0 ± 1.8 | | 5.4 ± 4.4 | | 0.002 | | -3.5 | | -63.7 | | | + | |
|  |  |  |  | Hip-knee-ankle angle (°) | 1.1 ± 0.9 | | 2.3 ± 2.0 | | 0.018 | | -1.2 | | -50.7 | | | + | |
|  |  |  |  | Medial proximal tibial angle (°) | 1.0 ± 0.6 | | 1.5 ± 1.0 | | 0.068 | | -0.5 | | -31.8 | | | (+) | |
|  |  |  |  | Correction angle (°) | 0.7 ± 0.6 | | 0.6 ± 0.4 | | 0.912 | | 0.0 | | 3.1 | | | (-) | |
|  |  |  |  | Posterior tibial slope angle (°) | 1.3 ± 1.3 | | 2.0 ± 2.0 | | 0.244 | | -0.1 | | -34.0 | | | (+) | |
|  | Zhu (2022)^67^ | 96 | ? | Hip-knee-ankle difference (°) | 0.6 ± 1.0 | | 2.6 ± 2.0 | | <0.001 | | -2.0 | | -76.9 | | | + | |
|  |  |  |  | Posterior tibial slope difference (°) | 1.6 ± 1.6 | | 2.9 ± 2.0 | | 0.006 | | -1.3 | | -44.8 | | | + | |
| Total shoulder arthroplasty | Boekel (2023)^68^ † | 47 | + | Guidewires placed within 2 mm of the planned position in superior/inferior plane (n, [%]) | 22 (91.7) | | 14 (14.0) | | 0.01 | | 30.8 | | 50.6 | | | + | |
|  |  |  |  | Guidewires placed within 2 mm of the planned position the AP plane (n, [%]) | 20 (83.3) | | 18 (78.3) | | 0.66 | | 5.1 | | 6.5 | | | (+) | |
|  | Hendel (2020)^69^ | 31 | + | Deviation in total offset (mm) | 2.4 ± 1.6 | | 3.4 ± 1.8 | | 0.11 | | -1.0 | | -29.4 | | | (+) | |
|  |  |  |  | Anteroposterior offset (mm) | 1.0 ± 0.9 | | 1.9 ± 1.4 | | 0.06 | | -0.9 | | -47.4 | | | (+) | |
|  |  |  |  | Medial-lateral offset (mm) | 1.0 ± 0.9 | | 1.9 ± 1.0 | | 0.012 | | -0.9 | | -47.4 | | | + | |
|  |  |  |  | Superior-inferior offset (mm) | 2.0 ± 1.5 | | 2.3 ± 2.1 | | 0.64 | | -0.3 | | -13.0 | | | (+) | |
|  |  |  |  | Deviation in version (°) | 4.3 ± 4.5 | | 6.9 ± 4.4 | | 0.11 | | -2.6 | | -37.7 | | | (+) | |
|  |  |  |  | Deviation in inclination (°) | 2.9 ± 3.4 | | 11.6 ± 7.0 | | <0.0001 | | -8.7 | | -75.0 | | | + | |
|  |  |  |  | Deviation in roll (°) | 6.5 ± 5.1 | | 10.2 ± 9.7 | | 0.13 | | -3.7 | | -36.3 | | | (+) | |
|  |  |  |  | Deviation from plan least retroverted (°) | 7.0 ± 5.4 | | 3.2 ± 2.1 | | 0.14 | | 3.8 | | 118 | | | (-) | |
|  |  |  |  | Deviation from plan most retroverted (°) | 1.2 ± 2.0 | | 10.0 ± 4.4 | | <0.001 | | -8.8 | | -88.0 | | | + | |
| Distal radius osteotomy | Buijze (2018)^72^ | 40 | ? | Ulnar length variance (mm) | 1.5 ± 1.1 | | 1.2 ± 1.1 | | 0.343 | | 0.3 | | 25.0 | | | (-) | |
|  |  |  |  | Volar-dorsal angulation (°) | 4.1 ± 3.3 | | 7.4 ± 4.0 | | 0.04 | | -3.3 | | -44.6 | | | + | |
|  |  |  |  | Radial inclination (°) | 2.2 ± 2.3 | | 4.9 ± 4.0 | | 0.028 | | -2.7 | | -55.1 | | | + | |
| Distal humerus osteotomy | Hu (2020)^73^ | 35 | ? | Difference in carrying angle between operated and contralateral side (°) | 1.1 ± 1.2 | | 4.2 ± 2.3 | | <0.001 | | -3.1 | | -73.2 | | | + | |
| Femoral neck fracture repair | Wang (2021)^76^ | 60 | - | Shortest distance between cannulated screw and cortex (mm) | 3.0 ± 0.4 | | 5.4 ± 0.7 | | <0.001 | | -2.3 | | -43.5 | | | + | |
|  |  |  | |  |  | |  | |  | |  | | | | |  | |
|  |  |  | |  |  | |  | |  | |  | | | | |  | |
| 1. Surgery duration | | | | | | | | | | | | | | | | | |
| Application | Reference | N | Overall Risk of Bias | Description duration | Value in intervention group | | Value in control group | | P-value | | Absolute change (Δ) | | Relative change (Δ%) | | | Positive or negative  significant outcome for PSG | |
| Spinal fusion | Cecchinato (2019)^50^ | 29 | + | Total surgery duration (min) in mean | 422 | | 423 | | >0.05 | | -1 | | -0.2 | | | (+) | |
|  | Zhang (2020)^52^ | 40 | ? | Total surgery duration (min) | 66.7 ± 6.8 | | 95.5 ± 7.2 | | <0.01 | | -28.8 | | -30.2 | | | + | |
|  | Feng (2012)^54^ | 12 | ? | Total surgery duration (min) | 171.7 ± 19.4 | | 175.8 ± 26.2 | | 0.76 | | -4.2 | | -2.4 | | | (+) | |
|  | Merc (2013)^56^ | 19 | - | Total surgery duration (min) | 143 ± 113 | | 176 ± 90 | | NR | | -33 | | -18.8 | | | (+) | |
| Total hip arthroplasty | Wang (2021)^57^ | 104 | + | Divided into four subgroups based Crowe’s classification for hip dysplasia (Crow I, II, III, IV) | | | | | | | | | | | | | |
|  |  | I |  | Total surgery duration (min) | 27.1 ± 3.4 | | 24.6 ± 3.4 | | 0.053 | | 2.5 | | 10.3 | | | (-) | |
|  |  | II |  | Total surgery duration (min) | 33.0 ± 6.5 | | 33.1 ± 4.4 | | 0.994 | | -0.1 | | -0.1 | | | (+) | |
|  |  | III |  | Total surgery duration (min) | 42.3 ± 4.2 | | 50.0 ± 1.5 | | 0.001 | | -7.7 | | -15.5 | | | + | |
|  |  | IV |  | Total surgery duration (min) | 61.4 ± 14.4 | | 70.5 ± 12.1 | | 0.151 | | -18.8 | | -12.8 | | | (+) | |
|  | Jin (2022)^58^ | 80 | + | Total surgery duration (min) | 78.4 ± 16.4 | | 74.2 ± 13.2 | | 0.583 | | 4.2 | | 5.7 | | | (-) | |
|  | Zhang (2011)^59^ | 20 | ? | Total surgery duration (min) | 118.6 | | 140.2 | | <0.05 | | -21.6 | | -15.4 | | | + | |
|  | Small (2014)^60^ | 36 | - | Total surgery duration (min) in median [IQR] | 95.0 [76.0-114.0] | | 88.0 [72.0-110] | | 0.46 | | 7 | | 8.0 | | | (-) | |
|  | Zhang (2011)^61^ | 22 | - | Total surgery duration (min) | 119.6 | | 125.3 | | NR | | -5.7 | | -4.5 | | | (-) | |
| High tibial osteotomy | Gao (2021)^66^ | 39 | + | Total surgery duration (min) | 109.4 ± 20.8 | | 131.7 ± 29.9 | | 0.014 | | -22.3 | | -16.9 | | | + | |
| Total shoulder arthroplasty | Boekel (2023)^68^ † | 47 | + | Total surgery duration (min) | 78.4 ± 16.3 | | 74.8 ± 10.3 | | 0.42 | | 3.6 | | 4.8 | | | (-) | |
| Percutaneous vertebroplasty | Hu (2021)^70^ | 36 | ? | Total surgery duration (min) | 19.4 ± 2.4 | | 27.8 ± 4.0 | | <0.05 | | -8.4 | | -30.2 | | | + | |
|  | Chen (2022)^71^ | 97 | - | Total surgery duration (min) | 57.1 ± 8.76 | | 75.4 ± 9.8 | | <0.001 | | -18.3 | | -24.3 | | | + | |
| Distal radius osteotomy | Buijze (2018)^72^ | 40 | ? | Total surgery duration (min) | 90.6 ± 32.3 | | 96.9 ± 34.3 | | 0.583 | | -6.3 | | -6.5 | | | (-) | |
| Correction of lower limb deformities | Fan (2022)^74^ | 55 | - | Total surgery duration (min) | 20.8 ± 2.4 | | 28.4 ± 2.35 | | <0.001 | | -7.6 | | -26.8 | | | + | |
| Acetabular osteotomy | Ma (2022)^75^ | 22 | - | Total surgery duration (min) | 114.7 ± 2.2 | | 150.4 ± 2.45 | | 0.0001 | | -35.7 | | -23.7 | | | + | |
|  |  |  | |  |  | |  | |  | |  | | | | |  | |
|  |  |  | |  |  | |  | |  | |  | | | | |  | |
| 1. Blood loss | | | | | | | | | | | | | | | | | |
| Application | Reference | N | Overall Risk of Bias | Description duration | Value in intervention group | | Value in control group | | P-value | | Absolute change (Δ) | | Relative change (Δ%) | | | Positive or negative  significant outcome for PSG | |
| Spinal fusion | Zhang (2020)^52^ | 40 | ? | Blood loss (mL) | 86.5 ± 5.2 | | 127.3 ± 5.1 | | <0.01 | | -40.8 | | -32.1 | | | + | |
|  | Feng (2012)^54^ | 12 | ? | Blood loss (mL) | 300.0 ± 89.4 | | 350.0 + 137.8 | | 0.315 | | -50 | | -14.3 | | | (-) | |
| Total hip arthroplasty | Wang (2021)^57^ | 104 | + | Divided into four subgroups based Crowe’s classification for hip dysplasia (Crow I, II, III, IV) | | | | | | | | | | | | | |
|  |  | I |  | Blood loss (mL) | 333.3 ± 149.4 | | 328.6 ± 133.8 | | 0.914 | | 4.7 | | 1.4 | | | (-) | |
|  |  | II |  | Blood loss (mL) | 361.5 ± 122.7 | | 382.1 ± 156.4 | | 0.706 | | -20.6 | | -5.4 | | | (+) | |
|  |  | III |  | Blood loss (mL) | 412.5 ± 83.5 | | 435.7 ± 102.9 | | 0.643 | | -23.2 | | -5.3 | | | (+) | |
|  |  | IV |  | Blood loss (mL) | 660.0 ± 206.6 | | 875.0 ± 173.6 | | 0.022 | | -215 | | -24.6 | | | + | |
|  | Jin (2022)^58^ | 80 | + | Intraoperative blood loss (mL) | 435.8 ± 73.3 | | 427.5 ± 69.8 | | 0.395 | | 8.3 | | 1.9 | | | (-) | |
|  |  |  |  | Total blood loss (mL) | 711.3 ± 159.5 | | 680.6 ± 148.2 | | 0.528 | | 30.7 | | 4.5 | | | (-) | |
|  | Zhang (2011)^59^ | 20 | ? | Intraoperative blood loss (mL) | 410.9 | | 480.6 | | <0.05 | | -69.7 | | -14.5 | | | + | |
|  | Zhang (2011)^61^ | 22 | - | Blood loss (mL) | 409.8 | | 480.6 | | NR | | -70.8 | | -14.7 | | | (+) | |
| High tibial osteotomy | Gao (2021)^66^ | 39 | + | Intraoperative blood loss (mL) | 50.6 ± 14.4 | | 97.8 ± 67.4 | | 0.003 | | -47.2 | | -48.2 | | | + | |
| Acetabular osteotomy | Ma (2022)^75^ | 22 | - | Intraoperative blood loss (mL) | 639.7 ± 5.0 | | 850.5 ± 5.3 | | 0.0001 | | -210.8 | | -24.8 | | | + | |
|  |  |  |  | 24h postoperative drainage volume (mL) | 231.2 ± 3.9 | | 324.3 ± 4.1 | | 0.0001 | | -93.1 | | -28.7 | | | + | |
|  |  |  |  | Postoperative blood transfusion rate (%) | 36.4 | | 63.6 | | 0.0001 | | -27.3 | | -42.9 | | | + | |
|  |  |  | |  |  | |  | |  | |  | | | | |  | |
|  |  |  | |  |  | |  | |  | |  | | | | |  | |
| 1. Radiation exposure | | | | | | | | | | | | | | |  |  |  |
| Application | Reference | N | Overall Risk of Bias | Description duration | Value in intervention group | | Value in control group | | P-value | | Absolute change (Δ) | | Relative change (Δ%) | | | Positive or negative  significant outcome for PSG | |
| Spinal fusion | Cecchinato (2019)^50^ | 29 | + | C-arm fluoroscopy shots (n) | 11 ± 9.87 | | 47.5 ± 15.3 | | 0.001 | | -36.5 | | -77 | | | + | |
|  |  |  |  | DAP (cGycm2) | 133.5 ± 59.6 | | 473.3 ± 448.3 | | 0.001 | | -339.8 | | -71.8 | | | + | |
|  |  |  |  | Fluoroscopy images (n) | 13.2 ± 8.7 | | 32 ± 20.9 | | 0.001 | | -18.8 | | -58.7 | | | + | |
|  |  |  |  | Exposure time (s) | 9.4 ± 2.9 | | 28.3 ± 27.7 | | NR | | -19.0 | | -66.8 | | | + | |
|  | Chen (2015)^51^ | 43 | ? | Fluoroscopy times per screw (s) | 0.5 ± 0.4 | | 1.2 ± 0.7 | | <0.05 | | -0.7 | | -58.3 | | | + | |
|  | Zhang (2020)^52^ | 40 | ? | Total radiation dosage (mSv) | 2.8 ± 0.5 | | 4.8 ± 0.8 | | <0.01 | | -2.07 | | -41.7 | | | + | |
|  |  |  |  | Radiation dosage until desired position (mSv) | 0.5 ± 0.1 | | 1.4 ± 1.4 | | <0.01 | | -0.9 | | -64.3 | | | + | |
| High tibial osteotomy | Gao (2021)^66^ | 39 | + | Radiation exposure (n) | 18.5 ± 4.8 | | 28.22 ± 4.28 | | 0.003 | | -9.7 | | -34.4 | | | + | |
| Percutaneous vertebroplasty | Hu (2021)^70^ | 36 | ? | Total fluoroscopy radiation dosage (mSv) | 4.9 ± 0.9 | | 7.9 ± 1.6 | | <0.05 | | -3.0 | | -38.0 | | | + | |
|  |  |  |  | Total fluoroscopy times (n) | 16.7 ± 2.9 | | 26.6 ± 5.3 | | <0.05 | | -9.9 | | -37.2 | | | + | |
|  |  |  |  | Fluoroscopy times /puncture point (n) | 1.8 ± 0.8 | | 5.2 ± 1.9 | | <0.05 | | -3.4 | | -65.4 | | | + | |
|  | Chen (2022)^71^ | 97 | - | Preoperative fluoroscopy/vertebra (n) | 1.04 ± 0.81 | | 6.12 ± 1.45 | | <0.001 | | -5.08 | | -83.0 | | | + | |
|  |  |  |  | Fluoroscopy time (sec) | 6.87 ± 1.31 | | 13.18 ± 1.78 | | <0.001 | | -6.31 | | -47.9 | | | + | |
| Distal radius osteotomy | Buijze (2018)^72^ | 40 | ? | Radiation time (sec) | 57.8 ± 37.7 | | 140.3 ± 101.4 | | 0.011 | | -82.5 | | -58.8 | | | + | |
| Correction of lower limb deformities | Fan (2022)^74^ | 55 | - | X-ray exposure times (n) | 3.50 ± 0.65 | | 4.70 ± 0.88 | | <0.001 | | -1.20 | | -25.53 | | | (+) | |
| Acetabular osteotomy | Ma (2022)^75^ | 22 | - | Fluoroscopy times (n) | 11.82 ± 0.42 | | 17.09 ± 0.39 | | 0.0001 | | -5.27 | | -30.48 | | | + | |
| *Values are given in means with standard deviation unless indicated otherwise. Absolute change (Δ) and relative change (Δ%) are based on mean or median values in the case of continuous variables. In the case of discrete variables, these are based on percentages. If authors used more than one decimal place in their study, values are rounded to one decimal place unless rounding results in zero. To calculate the absolute and relative change the unrounded values are used. “–” for significant negative differences, “+” for significant positive differences, “(–)” for insignificant negative differences, “(+)” for insignificant positive differences, and “=” for no difference between the two groups. “?” was used when it was unclear whether a result was positive or negative for the use of PSG.*  **This article performed an intention-to-treat and per protocol analysis but in this table we show the results of the intention-to-treat analysis.* † *In the control group virtual surgical planning was used. ~ Should not be determined because is preoperative measurement.*  *Abbreviations:*  *American Knee Society Score (AKSS), American Shoulder and Elbow Surgeons (ASES), Anterior Cruciate Ligament (ACL), Arbitraty Unit (AU), Constant Murley Score (CMS), Deep Venous Trombosis (DVT), Disabilities of the Arm, Shoulder and Hand (DASH), Dose Area Product (DAP), Harris Hip Score (HHS), International Knee Documentation Committee (IKDC), Inter quartile range (IQR), Japanese Orthopedic Association (JOA), Not Applicable (NA), Not Reported (NR), Not a Number (NaN), Oswestry Disability Index (ODI), Oxford Shoulder Score (OSS), Patient-Rated Wrist Evaluation (PRWE), Visual Analogue Score (VAS), Western Ontario and McMaster Universities Osteoarthritis Index (WOMAC).* | | | | | | | | | | | | | | | |  | |
